# TRIM56 Aggravates Cerebral Ischemia‐Reperfusion Injury via Inhibiting KLF4‐Activated Ferroptosis Signaling

**DOI:** 10.1002/advs.202509906

**Published:** 2025-11-10

**Authors:** Qiangping Wang, Shuang Li, Man Li, Wenke Zhou, Jianqing Zhang, Zhenfu Wu, Zhaoqi Mao, Juan Wan, Xinhao Tang, Baoping Zheng, Qiang Liu, Zhendong Li, Xiaobing Jiang, Qingping Wu, Youfan Ye, Haijun Wang

**Affiliations:** ^1^ Department of Neurosurgery Union Hospital Tongji Medical College Huazhong University of Science and Technology Wuhan 430022 China; ^2^ Department of Neurology Union Hospital Tongji Medical College Huazhong University of Science and Technology Wuhan 430022 China; ^3^ Department of Neurosurgery The First Affiliated Hospital of Xinxiang Medical University Weihui Henan 453100 China; ^4^ Department of Central laboratory Renmin Hospital of Wuhan University Wuhan Hubei 430060 China; ^5^ State Key Laboratory of North China Crop Improvement and Regulation Hebei Agricultural University Baoding Hebei 071000 China; ^6^ State Key Laboratory of New Targets Discovery and Drug Development for Major Diseases Gannan Innovation and Translational Medicine Research Institute Gannan Medical University Ganzhou 341000 China; ^7^ Department of Anesthesiology Union Hospital Tongji Medical College Huazhong University of Science and Technology Wuhan 430022 China; ^8^ Department of Ophthalmology Union Hospital Tongji Medical College Huazhong University of Science and Technology Wuhan Hubei 430022 China

**Keywords:** cerebral I/R injury, E3 ubiquitin ligase, ferroptosis, inflammation, TRIM56

## Abstract

Cerebral ischemia‐reperfusion (I/R) injury often causes significant neuronal damage, neurological deficits, and long‐term disability. This study investigates the role of tripartite motif‐protein 56 (TRIM56) in cerebral I/R injury and elucidates the underlying mechanisms. Here, a significant increase in TRIM56 expression in the human brain, mouse brain, and primary neurons after cerebral I/R injury is first detected. TRIM56 knockout mice exhibit reduced neurological deficits and a diminished inflammatory response, with TRIM56 overexpression intensifying these effects. Mechanistic investigations demonstrate that TRIM56 promotes neuronal ferroptosis by directly interacting with Krüppel‐like factor 4 (KLF4) and triggering its K48‐linked ubiquitination‐dependent degradation. Moreover, compound screening identifies farudodstat as a potential TRIM56 inhibitor to reduce I/R injury in vivo and in vitro. In conclusion, TRIM56 critically regulates neuronal damage during cerebral I/R injury, thereby presenting as a potential therapeutic target for reducing brain I/R injury. Novel therapeutic strategies inhibiting TRIM56 or its downstream signaling pathways may be developed to mitigate the devastating effects of I/R injury on neuronal survival and function.

## Graphical abstract

This study reveals that the E3 ubiquitin ligase TRIM56 exacerbates neuronal ferroptosis and brain damage by mediating K48‐linked ubiquitination and degradation of KLF4, leading to suppression of the xCT/GSH/GPX4 axis. Targeting TRIM56 alleviates cerebral ischemia‐reperfusion injury in vivo and in vitro, highlighting its therapeutic potential.

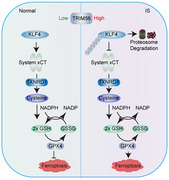

## 1 Introduction

Cerebral ischemia–reperfusion (I/R) injury is a critical medical condition characterized by an initial reduction of blood flow to the brain, followed by the restoration of circulation.^[^ ^1^ ^]^ This sequence of events can cause significant neuronal damage, neurological deficits, and long‐term disability.^[^ ^2^ ^]^ Understanding the underlying mechanisms of I/R injury is essential for developing effective therapeutic strategies that mitigate its impact.^[^ ^3^ ^]^ The pathophysiology of cerebral I/R injury involves a complex interplay of various cellular and molecular processes, including oxidative stress, inflammatory response, apoptosis, and necrosis.^[^ ^4^ ^]^ Inflammation is pivotal in this context, as activated microglia and infiltrating immune cells release pro‐inflammatory cytokines and chemokines that further exacerbate tissue damage.^[^ ^5^ ^]^

Ferroptosis is a significant cell death mechanism in ischemic injury.^[^ ^6^ ^]^ Distinct from the traditional forms of apoptosis and necrosis, it is characterized by the accumulation of lipid peroxides and its dependence on iron metabolism.^[^ ^6^, ^7^ ^]^ The induction of ferroptosis can cause rapid and irreversible neuronal damage, making it a critical area of investigation relevant to I/R injury.^[^ ^8^ ^]^ In particular, the relationship between tripartite motif‐containing proteins (TRIMs) and ferroptosis has attracted growing interest.^[^ ^9^ ^]^ Emerging evidence indicates that certain E3 ubiquitin ligases, which include TRIMs, can regulate ferroptosis by modulating the stability and activity of key proteins involved in this pathway.^[^ ^9^, ^10^ ^]^

The role of E3 ubiquitin ligases in the regulation of cellular responses to stress and injury has been significantly investigated.^[^ ^11^, ^12^, ^13^, ^14^ ^]^ TRIMs have been implicated in various biological processes, including immune responses, cell proliferation, and apoptosis.^[^ ^7^, ^15^, ^16^ ^]^ Specifically, TRIM56 (tripartite motif‐protein 56) contributes to the modulation of inflammatory responses and cell death pathways.^[^ ^17^, ^18^ ^]^ TRIM56 is upregulated in response to various stressors, including inflammatory signals and cellular damage.^[^ ^19^ ^]^ However, its specific involvement in cerebral I/R injury remains largely unexplored.

Elucidating the functions of TRIM56 during cerebral I/R injury would enhance our understanding of the cellular and molecular mechanisms involved in neuronal damage and recovery. Furthermore, clarifying its role in inflammation and ferroptosis would contribute to the development of innovative therapeutic approaches that can mitigate the devastating consequences of cerebral I/R.

## 2 Results

### 2.1 Upregulation of TRIM56 in Clinical and Experimental Models of Cerebral I/R Injury

**Figure 1:**
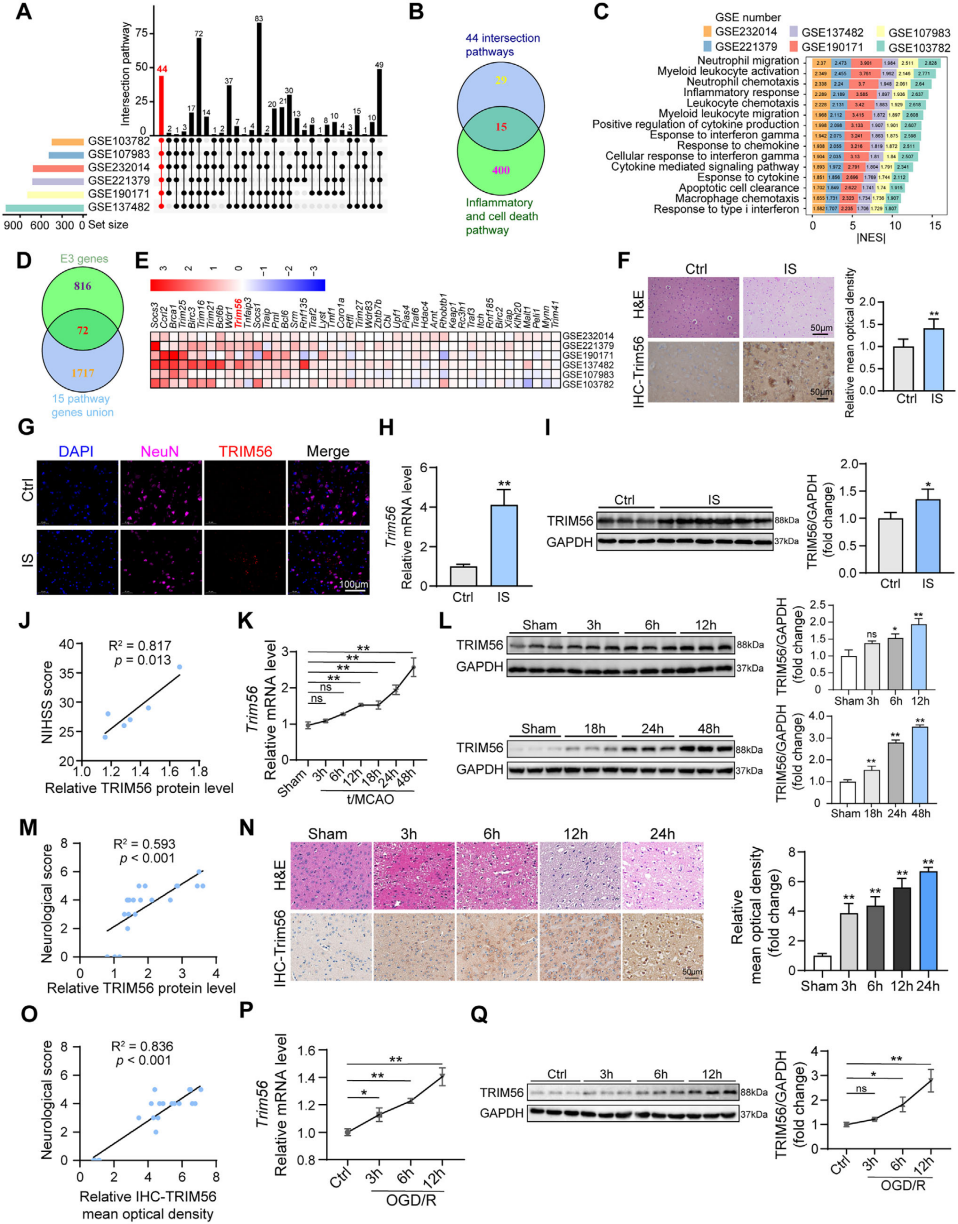
Upregulation of TRIM56 in clinical and experimental models of cerebral I/R injury. A) The intersection of enriched pathways from six publicly available transcriptomic datasets, which revealed 44 shared pathways associated with cerebral I/R injury. B) A Venn diagram analysis comparing “inflammatory cell death pathways” with the 44 shared pathways identified 15 key intersecting pathways. C) Normalized enrichment scores (NES) for the 15 intersecting pathways demonstrated significant activation in brain samples of ischemic stroke. D) The E3 ubiquitin ligases were screened in 15 pathways and 72 candidate E3 genes were cross‐matched. E) Heatmap analysis of the candidate E3 genes across multiple omics datasets revealed consistent upregulation of TRIM56 in ischemic brain samples. F) Representative images of H&E staining and IHC staining of TRIM56 in brain tissues of controlled and IS patient group (*n* = 3 for control, *n* = 3 for IS, scale bar = 50 µm). G) Representative images of IF staining of NeuN and TRIM56 in brain section of patients after cerebral I/R injury (*n* = 3 for control, *n* = 6 for IS t, scale bar = 100 µm). H) qRT‐PCR analysis of *Trim56* in controlled and IS patient group (*n* = 3 for control, *n* = 6 for IS patient). *Gapdh* served as a reference. I) Western blot analysis of TRIM56 in IS patients and controls (*n* = 3 for control, *n* = 6 for IS patient). J) Correlation analysis of NIHSS score and TRIM56 protein level in IS patients (*n* = 6). K) Quantification of mRNA expression level of *Trim56* in sham‐operated group and t/MCAO group at 3, 6, 12, 18, 24, and 48 h after modeling (*n* = 3 per group). *Gapdh* served as a reference. L) Western blot analysis of TRIM56 in sham‐operated group and t/MCAO group at 3, 6, 12, 18, 24, and 48 h after modeling (*n* = 3 per group). GAPDH served as a loading control. M) Correlation analysis of neurological score and TRIM56 protein level in sham‐operated and t/MCAO group (*n* = 21). N) Representative images and quantification of H&E staining and IHC staining of TRIM56 in mice (*n* = 5 per group). O) Correlation analysis of neurological score and mean optical density of TRIM56‐IHC in mice (*n* = 20). P) qRT‐PCR analysis of *Trim56* in primary neurons after OGD/R treatment and control (*n* = 3 per group). *Gapdh* served as a reference. Q) Western blot analysis of TRIM56 in primary neurons after OGD/R treatment and control (*n* = 3 per group). GAPDH served as a loading control. Notes: *P < 0.05; **P < 0.01; n.s.: not significant (*n* = 3 independent experiments).

First, gene set enrichment analysis (GSEA) was performed using six publicly available transcriptomic datasets (GSE190171, GSE103782, GSE107983, GSE232014, GSE221379, GSE137482) to systematically identify the key molecules involved in cerebral I/R injury. By intersecting the resulting pathways, 44 shared enriched pathways were identified (**Figure** **1**A). Subsequently, a Venn analysis comparing “inflammatory cell death pathways” with “intersection pathways” identified 15 key intersection pathways (Figure 1B). Examination of the normalized enrichment scores for these 15 pathways revealed that they were significantly activated in I/R‐stimulated brain samples and involved biological processes such as neutrophil migration, myeloid leukocyte activation, and inflammatory response (Figure 1C). Further cross‐referencing of genes within these 15 pathways against the E3 ubiquitin ligase family identified 72 candidate genes (Figure 1D). Among these candidates, TRIM56 stood out because of its consistent upregulation across multiple omics datasets (Figure 1E).

To confirm the effect of TRIM56 in CIRI, we collected brain tissues from ischemic stroke patients. Hematoxylin and eosin (H&E) staining revealed severe brain injury after I/R in IS patients. Moreover, immunohistochemistry (IHC) staining showed significant upregulation of TRIM56 in the ischemic brain samples (Figure 1F). Furthermore, immunofluorescent staining combining NeuN with TRIM56 demonstrated that TRIM56 was mainly expressed in neurons in the pathological state (Figure 1G). The results of RT‐PCR and western blot revealed significant upregulation of TRIM56 mRNA and protein levels in ischemic tissues compared with controls, indicating the potential involvement of TRIM56 in I/R pathophysiology (Figure 1H,I). More importantly, there was a significant correlation between TRIM56 protein level and NIHSS (National Institute of Health stroke scale, NIHSS) score in patients (Figure 1J). To further investigate the temporal expression of TRIM56, a t/MCAO mouse model was established to mimic cerebral I/R injury. Brain tissue samples were collected at various time points post‐reperfusion (3, 6, 12, 18, 24, and 48 h). RT‐PCR and western blot detection demonstrated a progressive increase in TRIM56 expression (Figure 1K,L). Similarly, the correlation analysis results of TRIM56 expression and animal neurological scores were consistent with the previous results (Figure 1M). In addition, IHC and subsequent correlation analysis revealed a systematic positive correlation between TRIM56 expression dynamics and neurological deficits (Figure 1N,O). These results firmly identified the potential bio‐marker value of TRIM56 in CIRI. Furthermore, TRIM56 expression was evaluated in an oxygen–glucose deprivation/reperfusion (OGD/R)‐induced cellular model using primary neurons, revealing a significant upregulation of TRIM56 in vitro (Figure 1P,Q). This cellular model further corroborates our findings from the analysis of clinical samples and the mouse model, highlighting the consistent induction of TRIM56 in response to ischemic stress. Collectively, these results provide compelling evidence of the upregulation of TRIM56 in both clinical and experimental settings of cerebral I/R, indicating its potential role as a critical mediator in the associated pathophysiological processes.

### 2.2 *Trim56* Knockdown Alleviated OGD/R‐Induced Inflammatory Responses In Vitro

**Figure 2:**
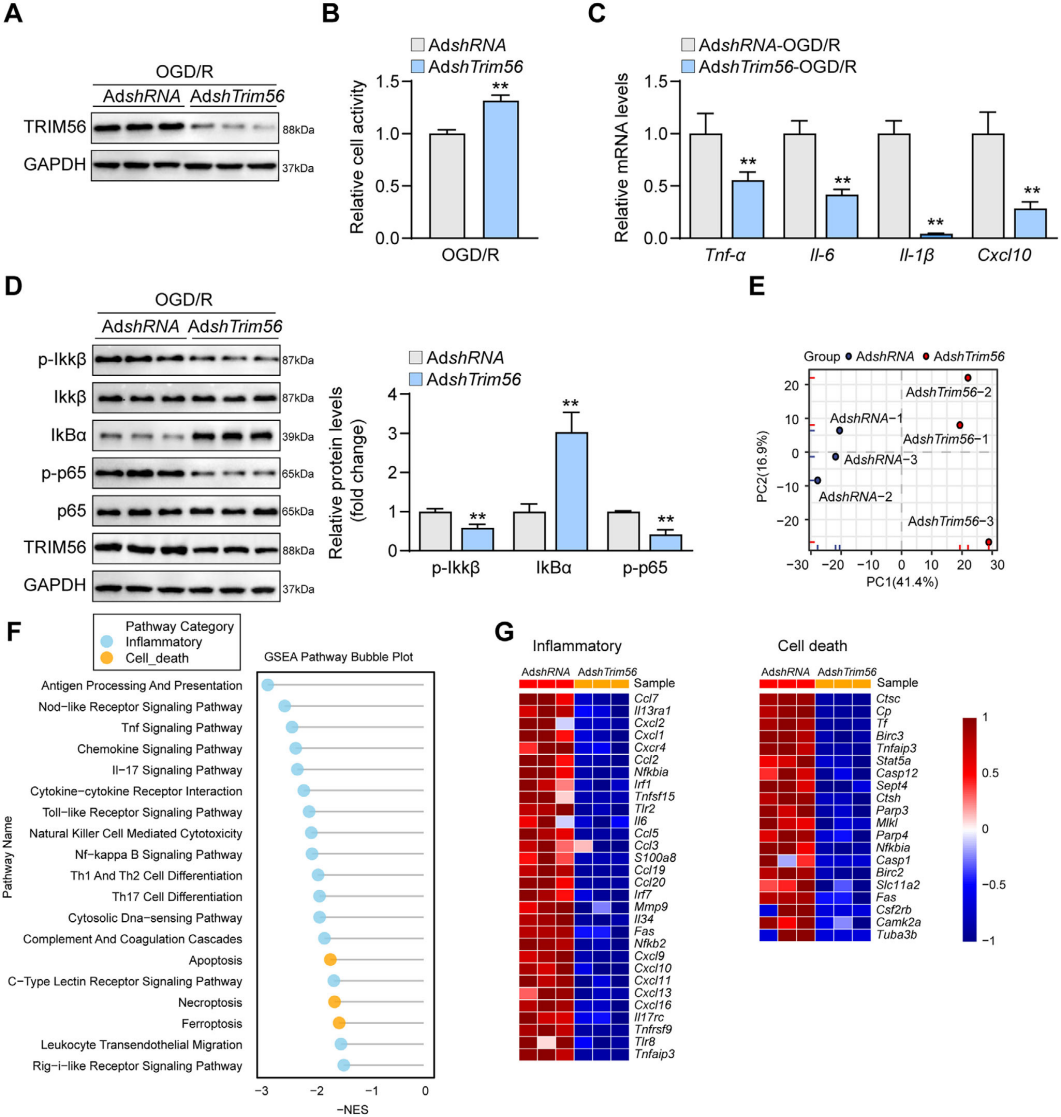
Trim56 knockdown alleviates OGD/R induced inflammatory responses in vitro. A) Western blot analysis of TRIM56 in primary neurons infected with TRIM56 knockdown viruses and controls after OGD/R treatment (*n* = 3 per group). GAPDH served as a loading control. B) Cell activity analysis of primary neurons infected with TRIM56 knockdown viruses and controls after OGD/R treatment (*n* = 5 per group). C) qRT‐PCR analysis of expressions of inflammatory cytokines in primary neurons infected with TRIM56 knockdown viruses and controls after OGD/R treatment (*n* = 4 per group). *Gapdh* served as a reference. D) Western blot analysis of NF‐κB proteins in primary neurons infected with TRIM56 knockdown viruses and controls after OGD/R treatment (*n* = 3 per group). GAPDH served as a loading control. E) Hierarchical clustering analysis of RNA‐seq results from primary neurons infected with TRIM56 knockdown viruses and controls after OGD/R treatment (*n* = 3 per group). F) GSEA basing on KEGG database of RNA‐seq results from primary neurons infected with TRIM56 knockdown viruses and controls after OGD/R treatment. G) Heatmap of DEGs related to inflammation and cell death (*n* = 3 per group). The asterisks indicate significant differences between the experimental group and the control group. *P < 0.05; **P < 0.01 (*n* = 3 independent experiments).

To explore the genetic function of TRIM56 in vitro, the TRIM56 knockdown adenoviruses were constructed, and an OGD/R model was established using primary neurons. The efficacy of TRIM56 knockdown viruses was first validated (**Figure** **2**A). The subsequent cell counting kit 8 (CCK‐8) cellular activity assay revealed that knocking down TRIM56 significantly improved cellular activity (Figure 2B), signifying the protective effects of TRIM56 knockdown. Furthermore, inflammatory cytokines produced by primary neurons harboring TRIM56 knockdown adenoviruses were expressed significantly lower than those by control cells (Figure 2C). Protein levels in typical NF‐κB signaling were synergistically altered, reducing the inflammatory responses (Figure 2D). To investigate the regulatory network of TRIM56, six TRIM56 knockdown groups and their corresponding control primary neurons after OGD/R modeling were subjected to RNA sequencing and bioinformatic analysis. Hierarchical clustering analysis revealed that the six groups were clearly divided into two subgroups, a control subgroup and a TRIM56 knockdown subgroup (Figure 2E), demonstrating good heterogeneity in the two subgroups. GSEA conducted based on the Kyoto encyclopedia of genes and genomes (KEGG) database identified 19 significantly enriched gene sets, which could be classified into the inflammation and cell death categories (Figure 2F). For a more detailed presentation, the heatmap showed that the differentially expressed genes (DEGs) related to inflammation and cell death were downregulated in the TRIM56 knockdown groups (Figure 2G). In summary, TRIM56 knockdown exerted a protective effect in primary neurons after OGD/R treatment.

### 2.3 TRM56 Overexpression Worsened OGD/R‐Induced Inflammatory Responses In Vitro

**Figure 3:**
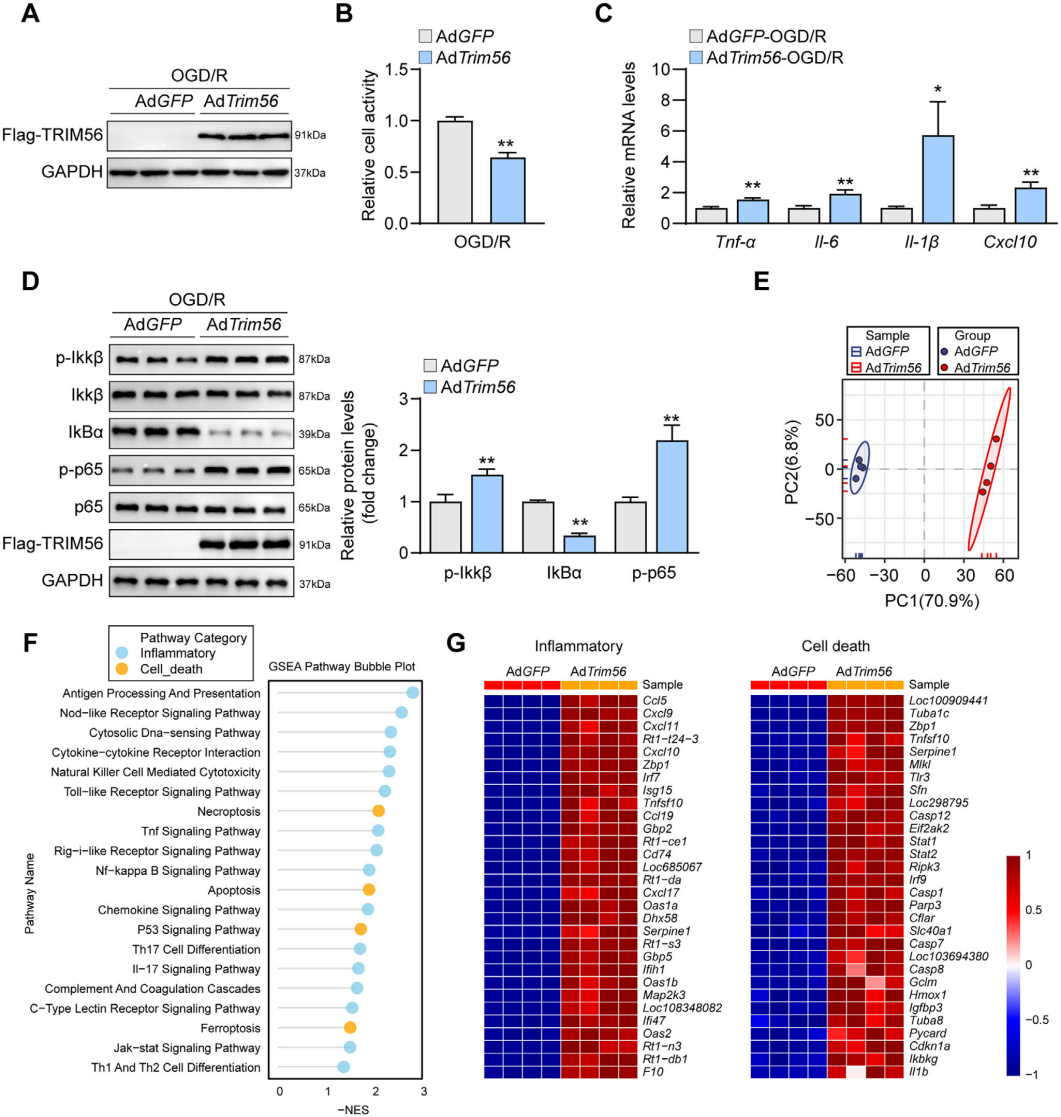
TRM56 overexpression worsened OGD/R‐induced inflammatory responses in vitro. A) Western blot analysis of TRIM56 in primary neurons infected with TRIM56 overexpression viruses and controls after OGD/R treatment (*n* = 3 per group). GAPDH served as a loading control. B) Cell activity analysis of primary neurons infected with TRIM56 overexpression viruses and controls after OGD/R treatment (*n* = 5 per group). C) qRT‐PCR analysis of expressions of inflammatory cytokines in primary neurons infected with TRIM56 overexpression viruses and controls after OGD/R treatment (*n* = 4 per group). *Gapdh* served as a reference. D) Western blot analysis of NF‐κB proteins in primary neurons infected with TRIM56 overexpression viruses and controls after OGD/R treatment (*n* = 3 per group). GAPDH served as a loading control. E) Hierarchical clustering analysis of RNA‐seq results from primary neurons infected with TRIM56 overexpression viruses and controls after OGD/R treatment (*n* = 3 per group). F) GSEA based on KEGG database of RNA‐seq results from primary neurons infected with TRIM56 overexpression viruses and controls after OGD/R treatment. G) Heatmap of DEGs related to inflammation (*n* = 3 per group). The asterisks indicate significant differences between the experimental group and the control group. *P < 0.05; **P < 0.01 (*n* = 3 independent experiments).

To obtain solid evidence of the functional effects of TRIM56 in neurons, the TRIM56 overexpression adenoviruses were constructed, and an OGD/R model was established using primary neurons. The efficacy of the TRIM56 overexpression viruses was validated by western blotting (**Figure** **3**A). CCK‐8 cellular activity assay showed that TRIM56 overexpression significantly inhibited cellular activity (Figure 3B), indicating that the overexpression of TRIM56 exerts detrimental effects. Furthermore, the inflammatory cytokines produced by primary neurons harboring TRIM56 overexpression adenoviruses were more significantly highly expressed than those by control cells (Figure 3C). Meanwhile, proteins in typical NF‐κB signaling were altered and exhibited higher inflammatory responses (Figure 3D). To elucidate the regulatory network of TRIM56, six TRIM56 overexpression groups and their corresponding control primary neurons after OGD/R modeling were subjected to RNA sequencing and bioinformatic analysis. Hierarchical clustering analysis revealed that the six groups were clearly divided into the control and TRIM56 overexpression subgroups (Figure 3E), demonstrating good heterogeneity in both subgroups. GSEA based on the KEGG database showed 20 significantly enriched gene sets, which could be classified into the inflammation and cell death categories (Figure 3F). The corresponding heatmap showed that the DEGs related to inflammation and cell death were upregulated in the TRIM56 overexpression groups (Figure 3G). In summary, TRIM56 overexpression exerted a detrimental role in primary neurons after OGD/R treatment.

### 2.4 *Trim56* Deficiency Alleviated Cerebral I/R Injury In Vivo

**Figure 4:**
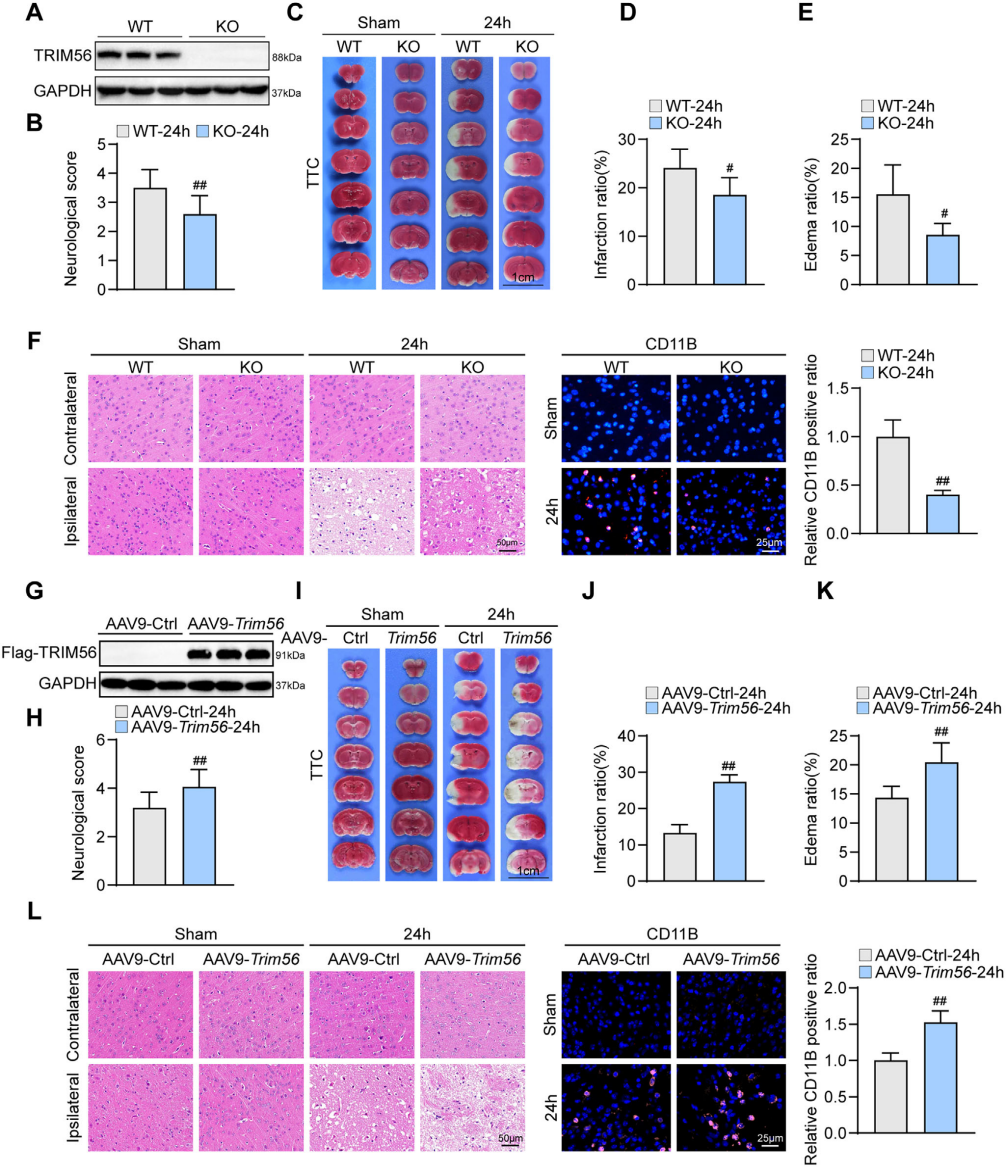
Genetic deficiency of TRIM56 alleviated cerebral I/R injury in vivo. A) Western blot analysis of TRIM56 in brain tissues of wild type and Trim56‐KO mice (*n* = 3 per group). B) The neurological score analysis of wild type and Trim56‐KO mice after cerebral I/R modeling (*n* = 15 per group). C–E) Representative images of TTC staining ((C); scale bar = 1 cm) and quantification results of infarction ratio (D) and edema ratio (E) in brains of wild type and Trim56‐KO mice after cerebral I/R modeling (*n* = 6 for infarction ratio; *n* = 4 for edema ratio). F) Representative images of H&E staining (scale bar = 50 µm) and CD11b staining (scale bar = 25 µm) in brain sections of wild type and Trim56‐KO mice after cerebral I/R modeling (*n* = 4 per group). G) Western blot analysis of TRIM56 in brain tissues of mice subjected to TRIM56 overexpression and control viruses’ injections (*n* = 3 per group). H) The neurological score analysis of mice injected with TRIM56 overexpression and control viruses (*n* = 15 per group). I–K) Representative images of TTC staining ((I); scale bar = 1 cm) and quantification results of infarction ratio (J) and edema ratio (K) in mice brains injected with TRIM56 overexpression and control viruses (*n* = 6 for infarction ratio; *n* = 5 for edema ratio). L) Representative images of H&E staining (scale bar = 50 µm) and CD11b staining (scale bar = 25 µm) in brain sections of mice after cerebral I/R modeling (*n* = 6 per group). The pound signs indicated significant differences between control group and gene‐editing group. ^#^ *P* < 0.05, ^##^ *P* < 0.01 (*n* = 3 independent experiments).

To evaluate the functions of TRIM56 in vivo, TRIM56‐KO and TRIM56 overexpression mice were established via the CRISPR/Cas9 and AAV9 systems, respectively. Successful gene knockout was confirmed by DNA sequencing (Figure S1, Supporting Information) and western blot analysis, which revealed the complete absence of TRIM56 expression in the brains of the KO mice compared with the wild‐type controls (**Figure** **4**A). Similarly, the KO mice or wild‐type controls were used to establish the cerebral I/R model and evaluated for neural damage. The neurological score of the Trim56‐KO mice was significantly lower than that of the control mice 24 h after cerebral I/R injury (Figure 4B), proving that TRIM56 exerts a detrimental effect in cerebral I/R injury. Furthermore, TTC staining, performed to quantitatively assess the infarct area in the brain tissue, revealed markedly decreased ischemic regions in the TRIM56 knockout mice, with a significantly reduced infarction and edema ratio compared with the wild‐type mice (Figure 4C–E). Histological analysis further corroborated these findings, showing a notable attenuation of pathological damage in the infarcted regions of Trim56‐KO mice (Figure 4F). Collectively, these results indicate that TRIM56 plays a detrimental role in cerebral I/R injury, as its knockout improved neurological outcomes and reduced infarct size. Therefore, TRIM56 is a potential therapeutic target in mitigating ischemic brain damage.

Furthermore, TRIM56 overexpression viruses adopting AAV9 were constructed, and their efficacy was validated in the brain tissues by western blotting (Figure 4G). Similar to previous experiments, the mice harboring control or TRIM56 overexpression viruses were used to establish a cerebral I/R model and evaluated for neural damage. Likewise, the neurological score of TRIM56 overexpression mice was significantly higher than that of control mice 24 h after cerebral I/R treatment (Figure 4H), further demonstrating that TRIM56 plays a detrimental role in cerebral I/R injury. Further TTC staining provided quantified evidence that the infarction and edema ratio was significantly higher in TRIM56 overexpression mice than in control mice (Figure 4I–K). H&E staining revealed that the brain injury in the infarcted area in wild‐type mice became more severe 24 h after cerebral I/R treatment due to TRIM56 overexpression. Furthermore, CD11b analysis revealed elevated microglial/macrophage activation in the TRIM56 overexpression group, signifying that increased inflammatory cell infiltration may exacerbate injury (Figure 4L).

### 2.5 Genetic Deficiency of *Trim56* Reduced t/MCAO‐Induced Inflammatory Responses In Vivo

**Figure 5:**
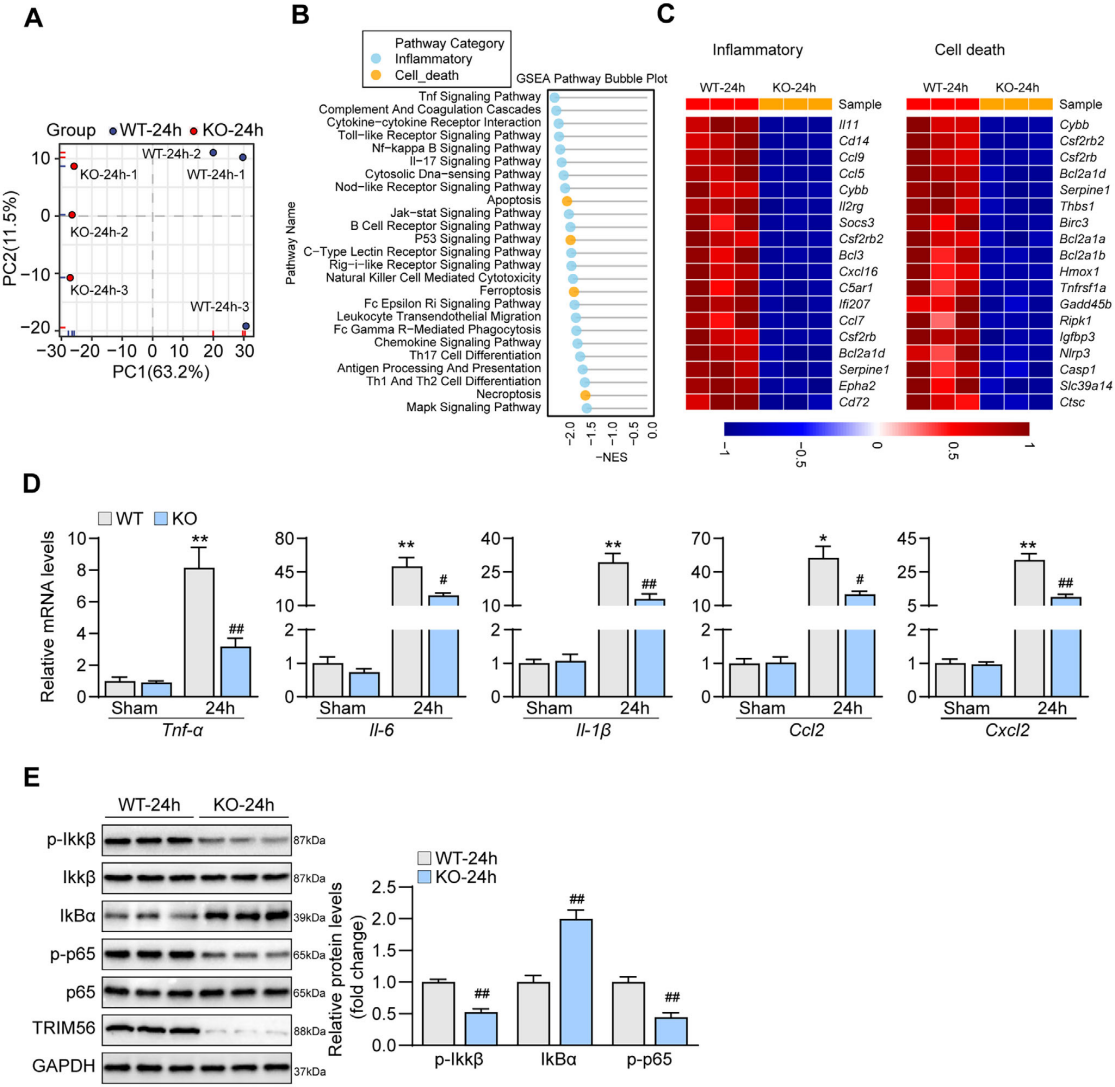
Genetic deficiency of *Trim56* reduced t/MCAO induced inflammatory responses in vivo. A) Hierarchical clustering analysis of RNA‐seq from brain tissues of wild type and Trim56‐KO mice t/MCAO modeling (*n* = 3 per group). B) GSEA based on KEGG database of RNA‐seq results from brain tissues of wild type and Trim56‐KO mice after t/MCAO modeling. C) Heatmap of DEGs related to inflammation and cell death (*n* = 3 per group). D) analysis of inflammatory cytokines in brains of wild type and Trim56‐KO mice after cerebral I/R modeling (*n* = 4 per group). *Gapdh* served as a reference. E) Western blot analysis of NF‐κB proteins in brains of wild type and Trim56‐KO mice after cerebral I/R modeling (*n* = 3 per group). GAPDH served as a loading control. The pound signs in this figure indicated significant differences between WT 24 h group and KO 24 h group. **P < 0.01, n.s.: not significant (*n* = 3 independent experiments).

To investigate the regulatory network of TRIM56, six groups of TRIM56‐KO mice and wild‐type controls after cerebral I/R modeling were subjected to RNA sequencing and bioinformatic analysis. Hierarchical clustering analysis revealed that the six groups were clearly divided into the control and TRIM56‐KO subgroups (**Figure** **5**A), demonstrating good heterogeneity in the two subgroups. GSEA based on the KEGG database revealed 25 significantly enriched gene sets, which were classified into the inflammation and cell death categories (Figure 5B). The more detailed heatmap showed that the DEGs related to inflammation and cell death were downregulated in the TRIM56‐KO groups (Figure 5C). Furthermore, the levels of inflammatory cytokines following cerebral I/R injury were significantly reduced in TRIM56‐KO mice compared with those in their wild‐type counterparts (Figure 5D). Proteins in typical NF‐κB signaling were synergistically altered, resulting in suppressed inflammatory responses (Figure 5E). All these results demonstrate that *Trim56* deficiency in the brain would reduce the in vivo inflammatory response (*n* = 3 independent experiments).

### 2.6 TRIM56 Exacerbated Cerebral I/R Injury by Promoting the Ferroptosis Pathway

**Figure 6:**
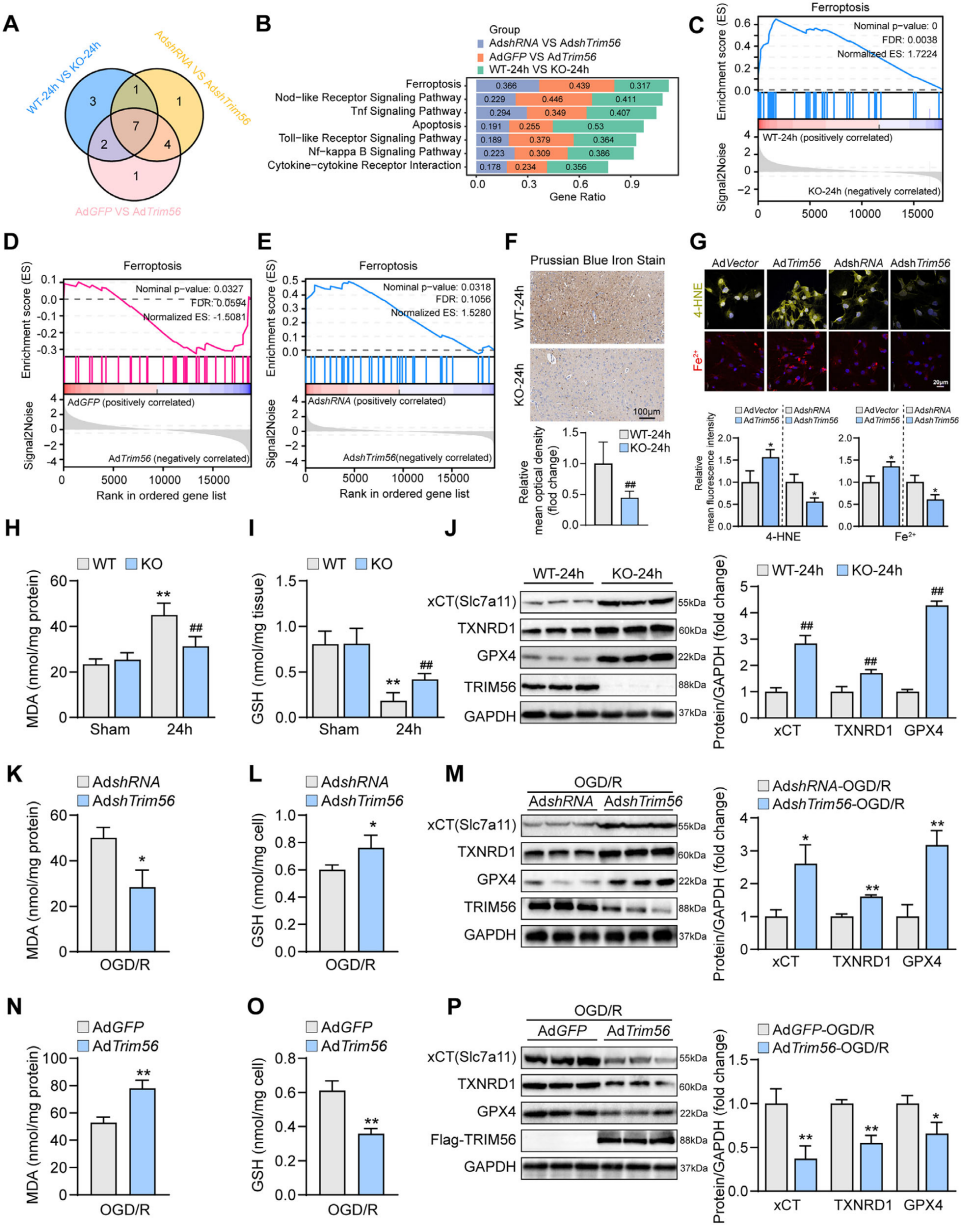
TRIM56 aggravates cerebral I/R injury by promoting ferroptosis pathway. A) Gene set enrichment analysis (GSEA) based on RNA sequencing results for Trim56‐KO and wild‐type mice brain samples after cerebral I/R modeling. B) Heatmap showing the differentially expressed genes associated with ferroptosis (*n* = 3 per group). C–E) The GSEA analysis based on RNA‐seq results of TRIM56 ‐KO mice (C), ‐overexpression (D), and ‐knockdown neurons (E). F) Prussian blue staining of brain sections from Trim56‐KO and wild type mice after cerebral I/R modeling (*n* = 6 per group). G) Representative images of 4‐HNE and Fe^2+^ staining in primary neurons after OGD/R stimulation (*n* = 3 per group). H,I) The content tests of MDA and GSH from Trim56‐KO and wild type mice brains after cerebral I/R modeling (*n* = 6 per group). J) Western blot (left) and quantification result (right) of ferroptosis‐related proteins in Trim56‐KO and wild‐type mice brains after cerebral I/R modeling (*n* = 3 per group). GAPDH served as a loading reference. K,L) The content tests of MDA and GSH in primary neurons infected with TRIM56 knockdown and control viruses after OGD/R treatment (*n* = 3 per group). M) Western blot (left) and quantification result (right) of ferroptosis‐related proteins in primary neurons infected with TRIM56 knockdown and control viruses after OGD/R treatment (*n* = 3 per group). GAPDH served as a loading reference. N,O) The content tests of MDA and GSH in primary neurons infected with TRIM56 overexpression and control viruses after OGD/R treatment (*n* = 3 per group). P) Western blot (left) and quantification result (right) of ferroptosis‐related proteins in primary neurons infected with TRIM56 overexpression and control viruses after OGD/R treatment (*n* = 3 per group). GAPDH served as a loading reference. The asterisks signs of this figure indicated significant differences between experimental group and control group (*P < 0.05, **P < 0.01), while the pound signs indicated significant differences between KO 24 h group and WT 24 h group (##*P* < 0.01) (*n* = 3 independent experiments).

Further GSEA analysis based on the RNA sequencing results of TRIM56 knockdown, TRM56 overexpression, and TRIM56 knockout revealed that ferroptosis was among the significant cell death signaling pathways regulated by TRIM56 (**Figure** **6**A,B). Furthermore, the ferroptosis pathway was significantly downregulated in the KO mice and knockdown primary neurons but upregulated in the overexpression primary neurons (Figure 6C–E), implying that TRIM56 activates ferroptosis signaling for ischemic stroke regulation. To verify the regulation of ferroptosis by TRIM56, Prussian Blue staining, Fe^2+^ probe, and 4‐hydroxynonenal (4‐HNE) staining were used to quantify the iron levels and lipid peroxidation products after cerebral I/R modeling in vivo and in vitro. The results revealed that the iron levels and 4‐HNE in the Trim56‐KO mice and Trim56‐knockdown neurons were significantly reduced compared with those in the control group (Figure 6F,G). Moreover, we performed western blot detection for several key proteins, which currently reported to be involved in progress of ferroptosis (Figure S2A, Supporting Information). Among these proteins, xCT‐TXNRD1‐GPX4 axis was significantly changed in TRIM56 overexpression neurons after OGD/R stimulation. Therefore, we focused on the TRIM56‐xCT/TXNRD1/GSH/GPX4 signaling pathway in further investigations. The decreased malonaldehyde (MDA) levels and increased glutathione (GSH) levels in the Trim56‐KO mice brain revealed that TRIM56 deficiency reduced the redox state in the brain, the predominant characteristic of ferroptosis, further implying the regulation of ferroptosis by TRIM56 (Figure 6H,I). Different pathways of ferroptosis‐related proteins were examined to elucidate how TRIM56 deficiency regulates ferroptosis. The pathway led by xCT, TXNRD1, and GPX4 was significantly upregulated under TRIM56 deficiency (Figure 6J). Thus, TRIM56 deficiency may exert protective effects on neurons from I/R injury by promoting ferroptosis‐inhibitory xCT/TXNRD1/GSH/GPX4 signaling.

Subsequently, the above hypothesis was tested using primary neurons. After OGD/R treatment, the MDA content severely decreased with TRIM56 knockdown, whereas the GSH content significantly increased, proving that the redox state was reduced by TRIM56 knockdown (Figure 6K,L). The expression levels of xCT, TXNRD1, and GPX4 were also elevated in TRIM56 knockdown neurons (Figure 6M). In contrast, TRIM56 overexpression promoted MDA production but decreased that of GSH, resulting in a predominantly oxidative state in neurons (Figure 6N,O). The expressions of xCT, TXNRD1, and GPX4 also synergistically decreased with TRIM56 overexpression (Figure 6P). Altogether, TRIM56 promoted ferroptosis by reducing xCT/TXNRD1/GSH/GPX4 signaling to exacerbate cerebral I/R injury, both in vivo and in vitro.

### 2.7 TRIM56 Promoted KLF4 Degradation to Inhibit xCT/TXNRD1/GSH/GPX4 Signaling

**Figure 7:**
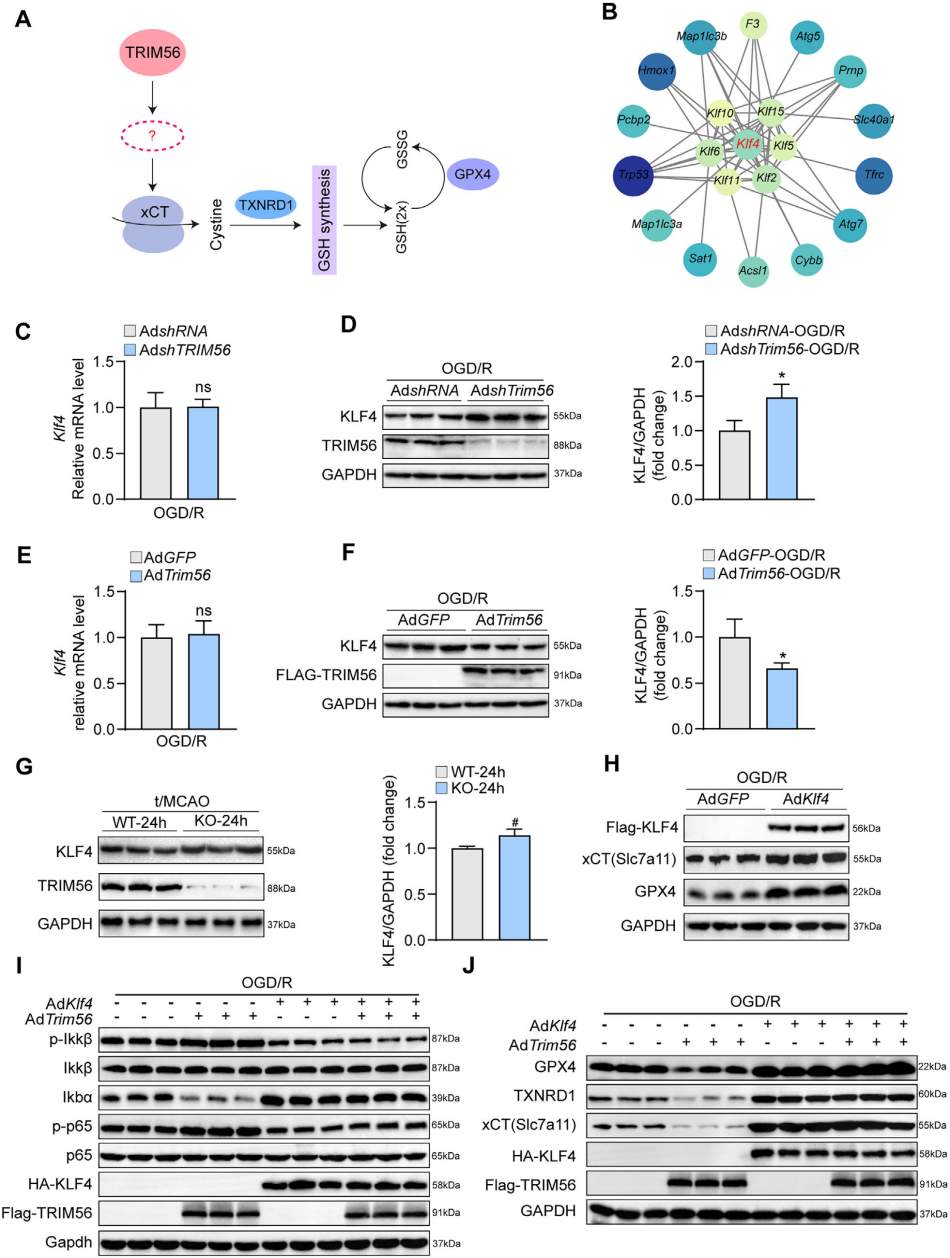
TRIM56 promoted KLF4 degradation to inhibit xCT/TXNRD1/GSH/GPX4 signaling. A) Hypothetical pattern diagram of regulation /GSH/GPX4 signaling pathway by TRIM56. B) Genomic analysis based on three RNA‐seq results. C) qRT‐PCR analysis of *Klf4* in primary neurons infected with TRIM56 knockdown or control viruses after OGD/R treatment (*n* = 4 per group). *Gapdh* served as a reference. D) Western blot (left) and quantification results (right) of KLF4 in primary neurons infected with TRIM56 knockdown or control viruses after OGD/R treatment (*n* = 3 per group). GAPDH served as a loading control. E) qRT‐PCR analysis of *Klf4* in primary neurons infected with TRIM56 overexpression or control viruses after OGD/R treatment (*n* = 4 per group). *Gapdh* served as a reference. F) Western blot (left) and quantification results (right) of KLF4 in primary neurons infected with TRIM56 overexpression or control viruses after OGD/R treatment (*n* = 3 per group). GAPDH served as a loading control. G) Western blot (left) and quantification results (right) of KLF4 in Trim56‐KO and wild type mice brains after t/MCAO surgery (*n* = 3 per group). GAPDH served as a loading control. H) Western blot results of xCT/GPX4 proteins in primary neurons infected with KLF4 overexpression or control viruses after OGD/R treatment (*n* = 3 per group). I,J) Western blot results of NF‐κB family and xCT/TXNRD1/GPX4 signaling proteins in primary neurons infected with different adenoviruses after OGD/R treatment (*n* = 3 per group). GAPDH served as a loading control. The asterisks signs in this figure indicated significant differences between indicated group and the control group. The pound signs indicated significant differences between WT 24 h group and KO 24 h group. * or #*P* < 0.05, **P < 0.01; n.s.: not significant (*n* = 3 independent experiments).

Since xCT, TXNRD1, and GPX4 did not interact with TRIM56 in primary neurons, we speculated that a mediator may facilitate between TRIM56 and xCT/TXNRD1/GSH/GPX4 signaling (**Figure** **7**A). First, our omics analysis revealed a potential association between KLF4 and TRIM56 (Figure 7B). Besides, KLF4 had been previously identified as an activator of the xCT/GSH/GPX4 axis and is thus a possible mediator in the regulation of TRIM56 on xCT/TXNRD1/GSH/GPX4 signaling.^[^ ^20^, ^21^ ^]^ This finding reveals critical insights into the influence of TRIM56 on neuronal function and survival via KLF4. Subsequently, we found that KLF4 expression increased in primary neurons after TRIM56 knockdown; however, the *Klf4* mRNA expression level remained unchanged (Figure 7C,D). In contrast, TRIM56 overexpression decreased KLF4 protein expression but not *Klf4* mRNA expression (Figure 7E,F). Similarly, KLF4 levels significantly increased in TRIM56 knockout mice brain tissues (Figure 7G). Therefore, KLF4, but not *Klf4* mRNA, is regulated by TRIM56.

To elucidate the role of KLF4 in regulating TRIM56‐mediated ferroptosis in cerebral I/R injury, the effects of KLF4 on xCT/TXNRD1/GPX4 signaling were analyzed. Western blot analysis revealed that KLF4 overexpression significantly upregulated xCT/TXNRD1/GPX4 signaling (Figure 7H). Furthermore, when TRIM56 and KLF4 were simultaneously overexpressed, NF‐κB signaling remained inactivated, indicating that TRIM56‐mediated activation of the inflammatory response in neurons was inhibited by KLF4 overexpression (Figure 7I). However, analysis of their effects on ferroptosis signaling after OGD/R treatment revealed that KLF4 promoted the accumulation of xCT, TXNRD1, and GPX4, whereas TRIM56 decreased it. When TRIM56 was simultaneously overexpressed with KLF4, xCT/TXNRD1/GPX4 signaling remained activated due to KLF4 overexpression, indicating that TRIM56‐mediated ferroptosis is inhibited by KLF4 overexpression (Figure 7J). These results demonstrate that KLF4 mediates the effects of TRIM56 in cerebral I/R injury. Therefore, KLF4 mitigated the detrimental effects of TRIM56 on cerebral I/R.

### 2.8 TRIM56 Interacted with KLF4 and Promoted Its Proteasome‐Mediated Degradation

**Figure 8:**
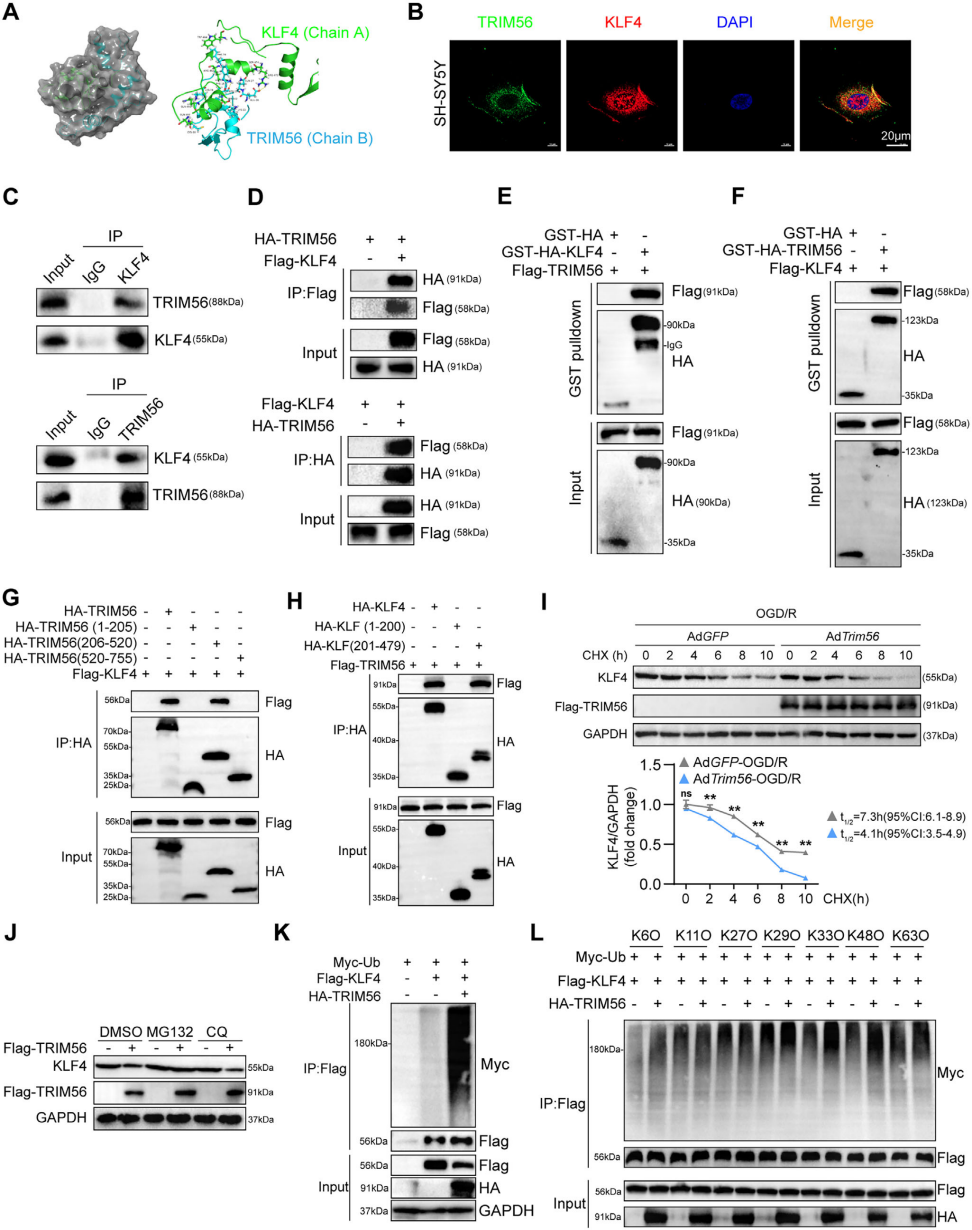
TRIM56 interacted with KLF4 and promoted its proteasome‐mediated degradation. A) The molecular docking results of TRIM56 and KLF4. B) The immunofluorescence staining of TRIM56 and KLF4 in SH‐SY5Y cells (Scale bar = 20 µm). C,D) Co‐IP assay of TRIM56 and KLF4 in primary neurons and HEK293T cells. E,F) GST‐pulldown assay of TRIM56 and KLF4 in HEK293T cells. G,H) Co‐IP assay adopting different truncations of TRIM56 and KLF4 in HEK293T cells. I) Primary neurons infected with GFP and Flag‐TRIM56 adenovirus were treated with the protein synthesis inhibitor cycloheximide (CHX; 50 µg mL^−1^) for the indicated times (*n* = 3 per group). J) Western bolt analysis of KLF4 protein level under different inhibitors when TRIM56 was overexpressed. GAPDH served as a loading control. K,L) Western blot analysis of total and different mutant ubiquitination level of KLF4. The asterisks signs in this figure indicated significant differences between Ad*Trim56* group and Ad*GFP* group. **P < 0.01, as; n.s.: not significant (*n* = 3 independent experiments).

In consideration of TRIM56 is an E3 ligase and KLF4 levels were decreased by TRIM56, we speculated that TRIM56 directly mediates KLF4 degradation. Thus, molecular docking studies were conducted to investigate the potential interaction between TRIM56 and KLF4. The results revealed a favorable binding mode between TRIM56 and KLF4, indicating a stable complex formation (**Figure** **8**A). To validate this result, we performed immunofluorescence (IF) staining of TRIM56 and KLF4 in SH‐SY5Y cells, which showed that TRIM56 and KLF4 spatially colocalized (Figure 8B). Subsequently, we performed co‐immunoprecipitation (Co‐IP) assays to investigate protein interactions between TRIM56 and KLF4, which revealed that TRIM56 could be coimmunoprecipitated with KLF4 in primary neurons and HEK293T cells (Figure 8C,D). Moreover, the GST pull‐down assay revealed that TRIM56 could directly interact with KLF4 (Figure 8E,F). Truncation assays further showed that the amino acids covering 206–520 of TRIM56 and 201–479 of KLF4 mediated the interaction between TRIM56 and KLF4 (Figure 8G,H). Furthermore, we performed a cycloheximide chase experiment on primary neurons, revealing a significant decrease in KLF4 half‐life time in Ad*Trim56* group as compared with control group (4.1h(95%CI:3.5–4.9) vs 7.3h(95%CI:6.1–8.9)) (Figure 8I). To elucidate how TRIM56 regulated the stability of KLF4, we added MG132 (a proteasome inhibitor) and chloroquine ((CQ) a lysosome inhibitor) to TRIM56 overexpression neurons. TRIM56 overexpression‐mediated KLF4 degradation was only prevented by MG132, demonstrating that KLF4 degradation by TRIM56 was proteasome dependent (Figure 8J). The ubiquitin assay showed that TRIM56 promoted KLF4 sum ubiquitination (Figure 8K), while the mutant ubiquitin assay revealed that TRIM56 increased the KLF4 ubiquitination at the K6, K33, and K48 sites (Figure 8L). These results show that TRIM56 directly interacted with KLF4 and promoted its degradation through proteasome‐mediated ubiquitination at the K6, K33, and K48 sites.

### 2.9 Farudodstat Inhibited TRIM56 to Alleviate Ischemic Stroke In Vivo and In Vitro

**Figure 9:**
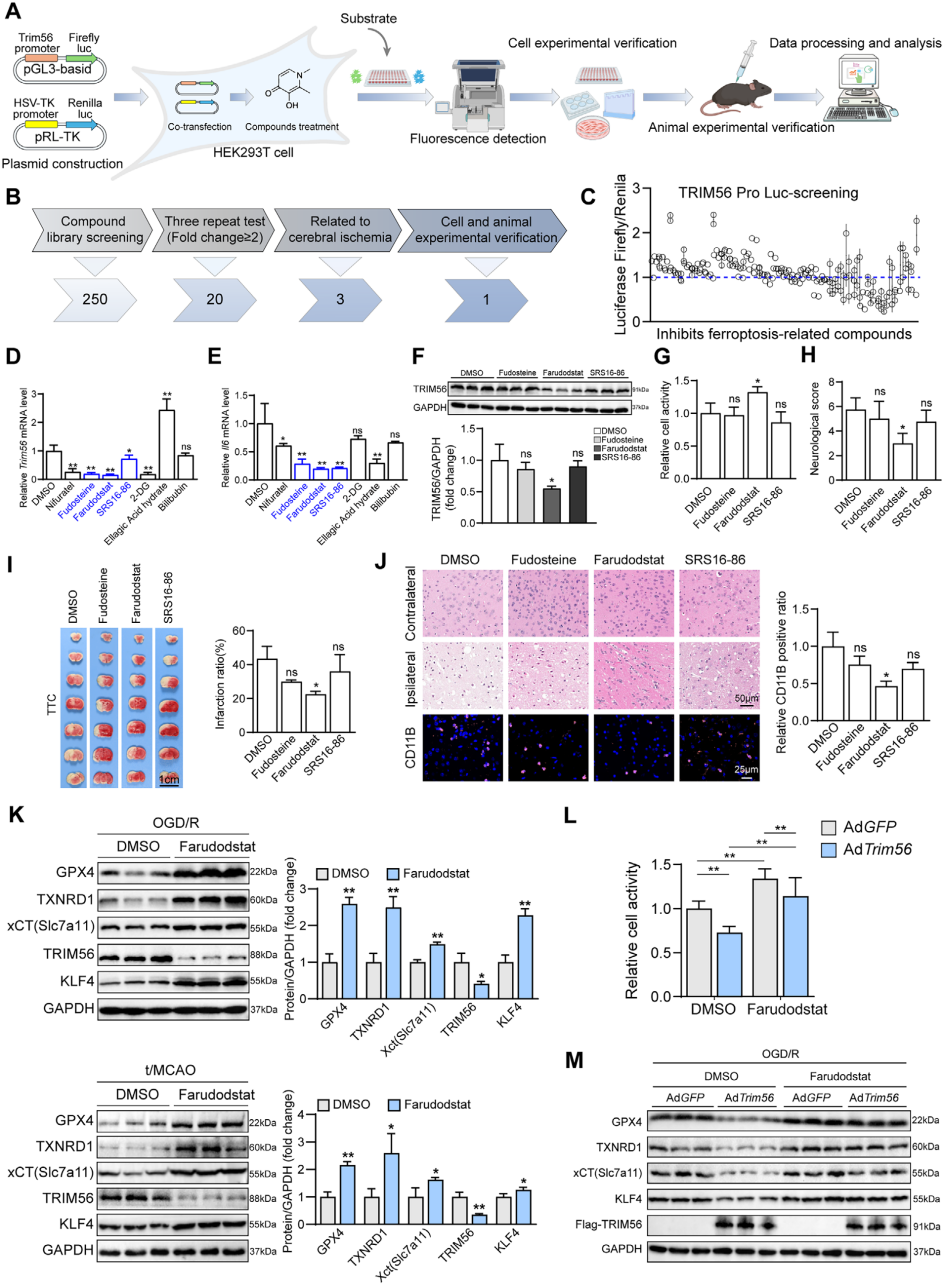
Farudodstat inhibited TRIM56 to alleviated ischemic stroke in vivo and in vitro. A) The work flow compound library screening using luciferase assay system. B) The summary chart of drug screening. C) The results of luciferase assay adopting compound library and TRIM56 promoter derived reporter (*n* = 3 independent experiments). D,E) qRT‐PCR analysis of *Trim56* and *IL‐6* in primary neurons treated with different compounds (*n* = 3 per group). *Gapdh* served as a reference. F) Western blot results of TRIM56 protein in primary neurons subjected to OGD/R treatment with Farudodstat, Fudosteine, SRS16‐86, or DMSO (*n* = 3 per group). GAPDH served as a loading control. G) Cell activity analysis of primary neurons subjected to OGD/R treatment with Farudodstat, Fudosteine, SRS16‐86, or DMSO (*n* = 4 per group). H) The neurological score analysis of mice subjected to cerebral I/R injury with Farudodstat, Fudosteine, SRS16‐86, or DMSO treatment (*n* = 4 per group). I) Representative images of TTC staining (scale bar = 1 cm) and quantification results of infarction ratio in brains of mice subjected to cerebral I/R injury with Farudodstat, Fudosteine, SRS16‐86, or DMSO treatment (*n* = 3 per group). J) Representative images of H&E staining (scale bar = 50 µm) and CD11b staining (scale bar = 25 µm) in brain sections of mice subjected to cerebral I/R injury with Farudodstat, Fudosteine, SRS16‐86, or DMSO treatment (*n* = 6 per group). K) Western blot results of TRIM56 and ferroptosis‐related proteins in primary neurons subjected to OGD/R treatment with DMSO or Farudodstat (*n* = 3 per group). GAPDH served as a loading control. L) Cell activity analysis of primary neurons infected with GFP or TRIM56 adenovirus subjected to OGD/R treatment with DMSO or Farudodstat treatment (*n* = 8 per group). M) Western blot results of TRIM56 and ferroptosis‐related proteins in wild type mice brains after t/MCAO surgery with or without Farudodstat addition (*n* = 3 per group). GAPDH served as a loading control. The asterisks signs in this figure indicated significant differences between indicated group and the control group. *P < 0.05, **P < 0.01, n.s.: not significant (*n* = 3 independent experiments).

To discover effective drugs for the development of a therapeutic strategy for ischemic stroke, we designed a work flow to identify drugs effective in lowering TRIM56 expression in HEK293T cells, with TRIM56‐promotor‐driven luciferase as a reporter (**Figure** **9**A). A Food and Drug Administration‐approved compound library was used to perform the screening. The compound library originally contained 250 chemicals; however, after three repeat assays, only 20 were left (Figure 9B). The luciferase assay revealed the inhibitory landscape of all chemicals in the ferroptosis compound library. Seven drugs exhibiting the most significant inhibitory effects were selected for further examination (Figure 9C). Ultimately, farudodstat, fudosteine, and SRS16‐86 could effectively reduce *Trim56* mRNA levels in primary neurons (Figure 9D). Synergistically, the mRNA expression of *Il‐6*, an inflammatory cytokine, showed a lowest level after treatment with farudodstat (Figure 9E). Moreover, we tested the effects of farudodstat, fudosteine, and SRS16‐86 on TRIM56 expression and cell viability, which found that TRIM56 expression was significantly reduced only by farudodstat, whereas cell viability increased (Figure 9F,G). To further investigate the effects of farudodstat, fudosteine, and SRS16‐86 on ischemic stroke, we injected farudodstat, fudosteine, and SRS16‐86 individually into mice 24 h before establishing the t/MCAO model. The results demonstrated that only farudodstat significantly alleviated cerebral I/R injury (Figure 9H–J). Furthermore, we tested the effects of farudodstat on TRIM56 and subsequent ferroptosis signaling and found that farudodstat significantly reduced TRIM56 expression, whereas KLF4 expression increased along with the expression of xCT, TXNRD1, and GPX4, indicating that farudodstat inhibited ferroptosis by inhibiting TRIM56 expression (Figure 9K). Ultimately, we performed rescue experiments to assess cell activity and the xCT–TXNRD1–GPX4 signaling pathway in TRIM56‐overexpressing cells following treatment with dimethyl sulfoxide (DMSO) and farudodstat. The results indicated that TRIM56 overexpression significantly reversed the effects of farudodstat on the xCT–TXNRD1–GPX4 axis (Figure 9L,M). In summary, our findings demonstrate that farudodstat effectively alleviates ischemic stroke by suppressing TRIM56 expression to inhibit ferroptosis. The simultaneous upregulation of KLF4 and key ferroptosis‐related proteins (xCT, TXNRD1, and GPX4) further supports the therapeutic potential of farudodstat. Thus, farudodstat is a promising candidate for the development of targeted therapies against ischemic stroke.

## 3 Discussion

Cerebral I/R injury is a complex pathological process that presents significant challenges in clinical settings, especially acute neurological deficits following strokes and transient ischemic attacks.^[^ ^4^, ^22^ ^]^ This study found that TRIM56 exacerbated neuronal damage during cerebral I/R injury, primarily through its effects on inflammation and ferroptosis.

The role of inflammation in cerebral I/R injury is well‐documented.^[^ ^23^ ^]^ Reperfusion activates a cascade of inflammatory responses that induce the recruitment of immune cells and the release of inflammatory mediators.^[^ ^24^, ^25^ ^]^ The exacerbation of these processes can in turn significantly worsen neuronal damage.^[^ ^25^ ^]^ TRIM56 enhanced these inflammatory responses, thereby significantly influencing the pathology of cerebral I/R injury. TRIM56 expression significantly increased in response to cerebral I/R, which is correlated with worsened neuronal injury. This observation aligns with those of previous studies demonstrating the upregulation of TRIM56 under various inflammatory conditions, particularly in ischemia diseases.^[^ ^26^ ^]^ TRIM56 upregulation indicates a potential compensatory mechanism that, paradoxically, contributes to tissue damage. This raises critical questions regarding the timing and regulation of TRIM56 expression during acute ischemic injury. Trim56‐KO mice exhibited significantly reduced neurological deficits and inflammatory responses compared with wild‐type controls. This protective effect demonstrates the detrimental role of TRIM56 in promoting neuroinflammation and apoptosis during cerebral I/R injury. Our findings are consistent with studies reporting that TRIMs can modulate inflammatory signaling pathways, such as the NF‐κB and MAPK signaling pathways, thereby potentially enhancing the production of pro‐inflammatory cytokines, such as TNF‐α and IL‐1β.^[^ ^10^, ^27^, ^28^ ^]^

One of the most significant findings in this study is the confirmation of ferroptosis as a major mechanism through which TRIM56 exacerbates cerebral I/R injury. Ferroptosis, which involves the accumulation of lipid peroxides and iron‐dependent cell death, has recently been recognized as a critical pathway in various neurological disorders.^[^ ^29^, ^30^ ^]^ Several signaling pathways participate in ferroptosis to determine cell fate, such as the xCT–GSH–GPX4 axis, an essential defense pathway.^[^ ^7^, ^31^, ^32^ ^]^ In cerebral I/R injury, hypoxia directly suppresses xCT activity, thereby triggering intracellular GSH depletion by blocking cystine uptake.^[^ ^31^, ^33^, ^34^, ^35^ ^]^ This loss of GSH inactivates GPX4, the GSH‐dependent enzyme.^[^ ^32^, ^33^ ^]^ The subsequent failure to reduce phospholipid hydroperoxides allows for their pervasive accumulation in cellular membranes, driving the execution of ferroptosis and resultant neuronal death.^[^ ^31^, ^32^, ^33^, ^34^, ^35^ ^]^ Targeting this pathway has proven to be a validated neuroprotective strategy.^[^ ^34^, ^36^ ^]^ Previous studies have confirmed that KLF4 acts as a promoter to reduce ferroptosis in Alzheimer's disease, diabetic nephropathy, atherosclerosis, and esophageal squamous cell carcinoma via xCT/GSH/GPX4 axis, which provided firmly evidences for our study.^[^ ^14^, ^20^, ^37^, ^38^ ^]^

Certain E3 ubiquitin ligases, including those in the TRIM protein family, can influence ferroptosis by regulating the stability of key proteins involved in this process.^[^ ^10^, ^30^ ^]^ For example, TRIM59, TRIM21, TRIM16, and TRIM7 promotes ferroptosis by inhibiting the xCT‐GSH‐GPX4 axis in non‐alcoholic fatty liver disease, prostate cancer, rheumatoid arthritis and asthma, respectively.^[^ ^10^, ^39^, ^40^, ^41^ ^]^ However, only a few TRIM proteins were reported to participate in the progression of ferroptosis in neurological disorders. In glioma animal models, TRIM7 and TRIM26 inhibited ferroptosis by targeting NCOA4 and GPX4, respectively.^[^ ^42^, ^43^ ^]^ Meanwhile, TRIM28 promoted ferroptosis by downregulating GSK3B in neuropathic pain.^[^ ^44^ ^]^ Remarkably, this study reported a novel finding that a TRIM protein act as a regulator of ferroptosis in CIRI. These findings expand the existing paradigm, providing new biological evidence and targets for understanding the progression of CIRI. The relationship between TRIM56 and ferroptosis is a worthy target of future investigations. The ability of TRIM56 to inhibit GPX4 or exhibit other protective mechanisms could explain the increased susceptibility to ferroptosis observed in our TRIM56 overexpression models.

The implications of our findings are profound. Elucidating the role of TRIM56 in cerebral I/R injury would enable the development of therapeutic interventions for mitigating ischemic brain damage. Targeting TRIM56 or its downstream pathways is a potential novel strategy for protecting neurons during acute ischemic events. Pharmacological TRIM56 inhibitors or other small molecules that disrupt the interaction of TRIM56 with key signaling pathways may offer potential therapeutic benefits. Farudodstat, a DHODH inhibitor, inhibits protein synthesis by activating the AP‐1 transcription factor.^[^ ^45^ ^]^ The utility of farudodstat as a potential inhibitor of I/R injury was first identified through compound library screening and functional validation. Furthermore, exploring the role of ferroptosis in I/R injury emphasizes the need for further studies into the use of ferroptosis inhibitors as a complementary approach to traditional neuroprotective strategies. In this context, the utility of compounds such as ferrostatin‐1 and liproxstatin‐1, which are potential ferroptosis inhibitors, to alleviate neuronal damage in cerebral I/R could be explored using cerebral I/R injury models. Combining these approaches may enhance neuroprotective strategies and improve functional recovery following ischemic events.

Although our study provides valuable insights into the role of TRIM56 in cerebral I/R injury, it has several limitations that warrant consideration. First, although the use of a mouse model is informative, it may not fully demonstrate the complexities of human cerebral ischemia. Future studies should validate our findings in larger animal models and eventually in clinical settings. Furthermore, we acknowledge that ferroptosis is a complex process regulated by multiple pathways, including iron metabolism and lipid peroxidation systems. Our current findings, while firmly establishing the TRIM56‐KLF4‐xCT/TXNRD1/GPX4 axis as a major mechanism, certainly do not exclude the possibility that TRIM56 may have additional discovered or undiscovered targets of ferroptosis. Investigating these potential alternative mechanisms represents a fascinating and important direction for future research, which would provide a more comprehensive understanding of multifaceted role of TRIM56 in cell death fate determination. Moreover, studies into the temporal dynamics of TRIM56 expression during I/R injury could provide insights into its potential as a therapeutic target. Finally, the potential interplay between TRIM56 and other forms of regulated cell death, such as apoptosis and necroptosis, were not investigated. Understanding the broader context of cell death mechanisms in cerebral I/R injury may help uncover a more comprehensive approach to neuroprotective therapies.

## 4 Conclusion 

In conclusion, our study identified TRIM56 as a significant mediator of cerebral I/R injury, primarily through its modulation of inflammatory responses and ferroptosis. Farudodstat reduced cerebral I/R injury by inhibiting TRIM56 expression, exhibiting its potential pharmacological value. TRIM56 exacerbated neuronal damage and thus emerges as a potential novel therapeutic target. Further elucidating the mechanisms by which TRIM56 functions would help uncover innovative strategies for mitigating the devastating effects of cerebral I/R, thereby ultimately improving outcomes for patients at risk of cerebral I/R injury. The development of effective therapies will require continuing research to unravel the complexities implicating TRIM56 in neuroinflammation and cell death. Furthermore, targeted pharmacological approaches that would harness the neuroprotective potential of TRIM56 should be developed.

## 5 Experimental Section

### Human Sample Collection

For the ischemic stroke group, brain tissues were collected from patients who underwent surgical decompression to correct intracranial hypertension after ischemic stroke. For the control group, brain tissues were collected from patients who underwent surgical decompression to correct intracranial hypertension caused by events other than ischemic stroke, such as traumatic brain injury.

Table S1 (Supporting Information) shows the clinical characteristics of patients.

### Construction of *Trim56* Knockout Mice

A sequence of guide‐RNA (sgRNA: 5′‐GGATGCTCATGCTGTCCGCCACAC‐3′) targeting *Trim56* was forecast using online CRISPR design tools (http://chopchop.cbu.uib.no/). The pUC57‐sgRNA (51132, Addgene) was used as the skeleton vector to construct the Trim56‐sgRNA expression vector. First, mix the purified products of the Cas9 expression vector pST1374‐Cas9 (44758, Addgene) and the sgRNA expression vector in vitro. Subsequently, the mixture was injected into the single‐cell fertilized C57BL/6 mouse ovum using the FemtoJet 5247 microinjection system (Eppendorf, Germany). The injected fertilized eggs were then transplanted into surrogate female mice. After 19–21 days of gestation, the F_0_ generation mice were obtained. To confirm the genetic phenotype, genomic DNA was extracted from toe tissues of the mice at two weeks after birth. The identification primers were as follows: Trim56‐KO‐F: 5′‐TGAGCAGCGATTTCCTAGCC‐3′; Trim56‐KO‐R: 5′‐GCATAAGTCGTCGGCACAGT‐3′. Male Trim56‐KO mice and their negative littermates (C57BL/6 background) aged 10–12 weeks and weighing 26–28 g were selected for further experiments. All mice were housed in the SPF Laboratory Animal Center of the Huazhong University of Science and Technology. The rearing conditions applied to all mice were a room temperature 22–24 °C, humidity 40%–70%, and 12‐h light/dark cycle.

### Adeno‐Associated Virus Serotype 9–*Trim56* Overexpression in Mice

For the construction of *Trim56* overexpression mice, the full length of the mouse *Trim56* cDNA (NM_201373.4) was packaged into recombinant adeno‐associated virus serotype 9 (AAV9‐hSyn) with *Flag* as the control. The viral solution (2 × 10^12^ vg mL^−1^, 5 µL/mouse) was slowly injected through the ventricles into mouse brain tissue after the mice were anesthetized by intraperitoneal pentobarbital administration (50 mg kg^−1^). Two weeks after the AAV9 injection, the animals were randomly assigned to either a sham operation group or a thromboembolic middle cerebral artery occlusion (t/MCAO) group. Blinded surgeries and subsequent analyses were performed subsequently.

### Construction of the Cerebral I/R Injury Mouse Model

Before the operation, the mice were anesthetized with 2.0% isoflurane and an oxygen/nitrous oxide mixture. Primarily, a longitudinal incision on the skin of the left cranial roof was made to expose the skull. The connective tissue was peeled from the skull surface. To record the cerebral blood flow, the regional cerebral blood flow (rCBF) was measured using a laser Doppler flowmetry instrument with a flexible probe affixed to the skull (1.5 mm posterior and 3–4 mm lateral to the bregma). The rectal temperature was maintained at 37 °C ± 0.5 °C using a homeothermic blanket. For the t/MCAO surgery, an 8‐0 silicon‐coated monofilament surgical suture was inserted into the left external carotid artery, advanced into the internal carotid artery, and wedged into the cerebral arterial circle to obstruct the origin of the middle cerebral artery. A >75% rCBF reduction indicated the interruption of blood flow in mice. The suture was withdrawn 45 min after ischemia, and a >70% return of the basal cerebral blood flow within 10 min of suture withdrawal confirmed reperfusion of the ischemic region. In contrast, the mice in which the suture was withdrawn immediately after the reduction in rCBF were used as the sham‐operated group.

### Neurological Function Scoring and Sampling

After 24 h of reperfusion, the neurological function score was evaluated using the modified Berderson scoring system (9‐point scale): 0 points, no symptoms of nerve damage; 1 point, the opposite forelimb curls up when lifting the tail, or cannot reach the affected forelimb completely; 2 points, the opposite shoulder is adducted when the tail is lifted; 3 points, flat push: resistance decreases when pushing to the opposite side; 4 points, moves spontaneously in all directions but only turns to the opposite side when taking off the tail; 5 points: turning in a circle or only turning to the opposite side when moving spontaneously; 6 points: involuntary movement, only when stimulated; 7 points: involuntary movement, no movement when stimulated; 8 points: death related to cerebral ischemia.^[^ ^46^ ^]^ After the evaluation, the mice were anesthetized with tribromoethanol (1.25%, 20 mL kg^−1^, intraperitoneal injection) and sacrificed. Brain tissues were collected for subsequent experiments.

### TTC Staining

The brain tissues were frozen at −20 °C for 30 min and cut into 1‐mm coronal sections. In general, 7 slices were cut (4 slices anterior and 3 slices posterior to the fontanel). The slices were immediately placed in 2% triphenyl tetrazolium chloride (TTC) staining solution and incubated at 37 °C for 10 min.

Variables were calculated as follows: 1) infarction percentage = (ipsilateral infarct area/contralateral brain volume/2) × 100%; and 2) edema ratio = (ipsilateral brain volume − contralateral brain volume)/contralateral brain volume × 100%. Further analysis of the infarct volume and proportion of edema was performed using Image J software (version 6.0, U.S. National Institutes of Health, Bethesda, MD).

### Pathological Analysis

Mouse brain tissue samples were immediately fixed in 10% neutral formalin, dehydrated, embedded in paraffin, cut into 5‐µm serial paraffin sections, and placed in an oven at 60 °C for 60 min. For H&E and Prussian Blue staining, hematoxylin (G1004, Sinopharm Group, China) and eosin (BA‐4024, BASO, China) dye or Prussian blue dye (234125, Sigma‐Aldrich, USA) was then added to the paraffin sections. The slices were then washed three times with distilled water, sealed with an ultraclean sealer (BA‐7004, BASO, China), and photographed under a light microscope (ECLIPSE 80i, Nikon, Japan).

For IHC and IF staining, the paraffin sections were first placed in an oven at 60 °C for 60 min and incubated with the corresponding primary antibody overnight at 4 °C, including CD11b (BM3925, Boster, USA), TRIM56 (ab154862, Abcam, UK), NeuN (ab177487, Abcam, UK), KLF4 (13673, ABclonal, China), and 4‐HNE (A26085, Abclonal, China). After washing with phosphate‐buffered saline, the sections were incubated with the secondary antibody for 1 h. The images were observed and photographed under a light microscope (Nikon, ECLIPSE 80i, Japan), fluoroscope (OLYMPUS, BX51, Japan), or confocal laser scanning microscope (Leica, TCS‐SP8, Germany), and the number of positive cells was analyzed using Image J software (version 6.0).

### Isolation, Culture, and Oxygen–Glucose Deprivation/Reperfusion Treatment of Cells

HEK293T cells (CRL‐3216, ATCC, USA) were cultured in Dulbecco's Modified Eagle Medium (DMEM, 11966025, Gibco) containing 10% fetal bovine serum (FBS‐S500, NEWZERUM, New Zealand) and 1% penicillin‐streptomycin antibody (15070063, Gibco). SH‐SY5Y cells (CRL‐2266, ATCC, USA) were cultured in DMEM/F12 medium (11320033, Gibco) containing 10% fetal bovine serum (FBS‐S500, NEWZERUM, New Zealand) and 1% penicillin‐streptomycin antibody (15070063, Gibco).

Cerebral cortices were collected from 1‐ to 2‐day‐old Sprague‐Dawley rats to obtain primary neurons. The brain tissues were cut into small pieces and digested with 10 mL of 0.125% trypsin (25300120; 25200072, Gibco) for 15 min at 37 °C. The digestion reaction was cultured in DMEM/F12 medium (11320033, Gibco) containing 10% fetal bovine serum (FBS‐S500, NEWZERUM, New Zealand) and DNA enzyme (11284932001, Roche). Clumps of cells and undigested tissue blocks were removed with a 100‐µm cell strainer. Subsequently, the cells were centrifuged at 1500 rpm for 5 min at 4 °C. The cell pellet was resuspended in DMEM/F12 medium containing 10% fetal bovine serum and 1% double antibody (15070063, Gibco). After their viability was determined, the cells were seeded into poly‐lysine (10 mg mL^−1^, Sigma)‐coated dishes and cultured in a 5% CO_2_ incubator at 37 °C for 3 h. Subsequently, the medium was replaced with neurobasal medium (10888022, Gibco) containing 2% B27 (17504044, Gibco) until the cells developed synapses and acquired a tadpole shape. The medium was protected from light and replaced every 3 d. The experiments were conducted after at least 7 days of culture.

The OGD/R model was constructed to mimic cerebral I/R in vitro. Briefly, the neurons were cultured in DMEM without glucose (11966025, Gibco) under hypoxic conditions (94% N_2_, 5% CO_2_, and 1% O_2_) for 3 h. Subsequently, the medium was replaced with neurobasal medium containing 2% B27 and cultured under normal oxygen conditions for 6 h. The cells in the control group were not subjected to hypoxia or glucose‐deprived medium.

### Cell Counting Kit 8 Assay

The activity of the primary neurons was detected using a CCK‐8 assay kit (44786, Dojindo) according to the manufacturer's guidelines. The primary neurons infected with the corresponding adenovirus were inoculated into 96‐well plates (167008, Thermo Fisher Scientific). Three cell‐free wells served as blank controls. After OGD/R treatment, the medium was replaced with the CCK‐8 reaction solution and incubated at 37 °C for 2 h. The absorbance value (optical density) at 450 nm was measured, and the data were recorded.

### Detection of Ferroptosis Levels

The MDA, GSH, and Fe^2+^ levels in brain tissues and primary neurons were analyzed using a lipid peroxidation MDA assay kit (S0131, Beyotime, China), GSH assay kits (S0053, Beyotime), and FerroOrange probe kit (F374, DOJINDO, Japan), respectively.

### RNA Sequencing

Mice brain samples and primary neurons were collected after t/MCAO modeling and OGD/R treatment. Then, the total RNA from the samples was extracted for further sequencing. First, the sequencing library was constructed using the MGI Tech kit (Shenzhen, China). Subsequently, a BGI sequencer (MGISEQ‐2000 platform) was used for sequencing, and raw reads were obtained. SOAPnuke software (version 2.0.7) was used to filter out reads with low‐quality, contaminated joints and high levels of unknown bases. The clean reads were then aligned to the reference genome using Hierarchical Indexing for Spliced Alignment of Transcripts 2 (HISAT2) software (version 2.1.0). Gene expression levels were calculated using StringTie software (version 1.3.3b). Finally, DESeq2 (version 1.2.10) was used to calculate the DEGs between the two groups of cells. The unweighted pair group method with arithmetic mean approach was adopted to perform hierarchical clustering analyses. Visualization of the clustering tree was achieved using the crust function of the R software.

### Kyoto Encyclopedia of Genes and Genomes Enrichment Analysis

KEGG is a comprehensive database that integrates genomic, chemical, and systemic functional information that can be used to classify differential genes into different biological pathways according to their annotation results and official classification. Here, the R software was used to perform enrichment analysis of all differential genes. Pathways with *P* < 0.05 were considered as significant enrichment pathways.

### Gene Set Enrichment Analysis

GSEA is specifically performed to arrange all genes from high to low according to expression level. The enrichment score is then calculated from the distribution of KEGG gene sets in the above gene list to obtain the overall expression change of these gene sets. GSEA was performed using the Java GSEA (version 3.0) platform, and gene sets with *P* values < 0.05 and false discovery rate (FDR) < 0.25 were considered statistically significant.

### Adenovirus Construction

The rat full‐length *Trim56* (NM_001399685) and *Klf4* (NM_053713.1) coding sequences were correctly cloned and assembled into the replication‐deficient adenovirus vector driven by the CMV promoter (Ad*Trim56*) to achieve TRIM56 or KLF4 overexpression. The adenovirus with *GFP* overexpression served as the control (Ad*GFP*). The selected short hairpin RNA targeting *Trim56* (Adsh*Trim56*) was subcloned into the above‐described adenovirus to knock down *Trim56*, and an AdshRNA adenovirus was used as a control. A 150–200 particle/cell multiplicity of infection was adopted to determine the adenovirus usage amount, and the infection and effective expression times determined were 8 and 24 h, respectively.

### Quantitative Real‐Time Polymerase Chain Reaction

Brain tissues and cells were lysed with TRIzol reagent (15596‐026, Invitrogen, USA). The HiScript II 1st Strand cDNA Synthesis Kit (R211‐01, Vazyme, China) was used to complete the first strand synthesis. The polymerase chain reaction (PCR) system was composed of SYBR Green PCR Master Mix (04887352001, Roche, Switzerland), primers, and cDNA. The LightCycler 480 System (Roche, Switzerland) was used as per the established protocol for quantification and subsequent analysis.

The primers used in this study are listed in Table S2 (Supporting Information).

### Western Blot

Radioimmunoprecipitation assay buffer (65 × 10^−3^ m Tris‐HCl [pH 7.5], 150 × 10^−3^ m NaCl, 1 × 10^−3^ m EDTA, 1% NP‐40 [Solarbio, N8030], 0.5% sodium deoxycholate, 0.1% sodium dodecyl sulfate [SDS]) formulated by ultrasonication was used for protein extraction from tissues, while the SDS lysis buffer (50 × 10^−3^ m Tris‐HCl [pH 6.8], 2% SDS, 10% glycerol) was used for protein extraction from cells. Protease inhibitors (04693132001, Roche, Switzerland) were included in the extraction buffers. A bicinchoninic acid protein assay kit (23225, Thermo Fisher Scientific, USA) was used for protein quantification, while SDS–polyacrylamide gel electrophoresis gels and polyvinylidene fluoride membranes (IPVH00010, Millipore, USA) were used for protein separation and transfer, respectively. Protein blocking was conducted using 5% skim milk for 1 h at room temperature, and the membranes were washed with 1× Tris‐buffered saline with Tween 20 buffer. The primary antibodies were incubated with the membranes at 4 °C overnight, while the corresponding secondary antibodies (115‐035‐003 or 111‐035‐003, Jackson ImmunoResearch Laboratories, USA) were incubated with the membranes at room temperature for 1 h. An enhanced chemiluminescence western blot substrate kit (BLWB021‐100ML, BioLight, China) and the ChemiDoc XRS+ Imaging System (Bio‐Rad, USA) were used to detect and visualize the proteins, respectively. The primary antibodies used in this study are listed in Table S3 (Supporting Information).

### Co‐immunoprecipitation and Ubiquitination Assay

Co‐IP was performed using HEK293T cells. The prepared cells were lysed in ice‐cold IP lysis buffer (20 × 10^−3^ m Tris‐HCl [pH 7.4], 150 × 10^−3^ m NaCl, 1 × 10^−3^ m EDTA, 1% NP‐40) containing protease inhibitors (04693132001, Roche, Switzerland). The cell lysates were centrifuged and subjected to IP using protein A/G agarose (AA104307, Bestchrom, China) beads with specified antibodies at 4 °C overnight. After washing, the beads were resuspended in 2× SDS loading buffer (100 × 10^−3^ m Tris‐HCl [pH 6.8], 4% SDS, 20% Glycerol and 0.03% bromophenol blue) and denatured at 95 °C for 10 min. The samples were then analyzed by western blot to determine the protein interactions.

### Glutathione‐S‐transferase Pull‐Down

HEK293T cells infected with the appropriate plasmids were collected and lysed in precooled glutathione‐S‐transferase (GST) lysis buffer (50 × 10^−3^ m Na_2_HPO_4_ [pH 8.0], 300 × 10^−3^ m NaCl, 1% Triton X‐100) containing protease inhibitors. The lysates of the bait and prey were centrifuged and incubated with GST beads at 4 °C for 3 h, respectively. The supernatants of the prey were collected and incubated at 4 °C overnight with the well‐washed conjugates of the GST beads and the baits. After washing, the final conjugates in the GST beads were denatured at 95 °C for 10 min and analyzed by western blot.

### Molecular Docking

The crystal structures of TRIM56 and KLF4 were obtained from the Protein Data Bank (PDB) database. The protein models used for docking were TRIM56_HUMAN (PDB ID: 5JW7) and KLF4_HUMAN (PDB ID: 6VTX). The HDOCK SERVER (http:// hdock.phys.hust.edu.cn/) was used for protein–protein molecular docking. The protein model was pretreated with PyMol 2.4 (removing water molecules and excess ligands and adding hydrogen atoms). The docking score, confidence score, and ligand root mean square deviation were used as the docking evaluation criteria. The model with the highest score was selected as the best docking model. Pymol 2.4 software was used to visualize the docking results.

### Compound Screening

The *Trim56* promoter was cloned and inserted into pGL3.1 to drive the expression of firefly luciferase, which served as a reporter. The reporter and sea cucumber luciferase plasmids were co‐transfected into HEK293T cells with the compound addition. Twenty‐four hours after transfection, the cells were dissolved with 1× passive lysis buffer (Dual‐Luciferase Reporter Assay System, Promega). LAR II and Stop & Glo Reagent were then added to collect the fluorescence signals from the multifunction reagent using GloMax 20/20 (Promega, USA). In the dual‐luciferase reporter assay, compounds that inhibited promoter activity by more than 20% were selected as candidates.

### Compound Treatment

The solvent was prepared by mixing 10% DMSO, 40% polyethylene glycol 300, 5% Tween 80, and 45% saline (0.9% NaCl) in the specified proportions. The solution was then vortexed before treatment. Twenty‐four hours before modeling, the suspension of the target compound at a dose of 50 mg kg^−1^ was administered by oral gavage to mice. Both the model and sham operation groups were given the same amount of solvent as the control.

### Statistical Analysis

The data are expressed as mean ± standard deviation. Statistical analyses were performed using SPSS 21.0 (SPSS Inc.). The normality test was performed using Shapiro–Wilk analysis. For data with a normal distribution, a two‐tailed Student's *t*‐test was used to compare the differences between the two groups. Differences among multiple groups were compared using one‐way analysis of variance followed by Bonferroni analysis (for data meeting homogeneity of variance) or Tamhane's T2 analysis (for data demonstrating heteroscedasticity). For data sets with a skewed distribution, the Mann–Whitney *U* test was used for comparisons between the two groups. The *p*‐value < 0.05 indicated statistical significance. All images shown without biological replicates are representative of a minimum of three independent experiments.

## Conflict of Interest

The authors declare no conflict of interest.

## Author Contributions

Q.W., S.L., and M.L. contributed equally to this work. H.W., Y.Y., and Q.W. designed and supervised this research. Q.W., S.L., M.L., Z.M., and Q.L. performed animal experiments; Q.W., S.L., Z.M., J.Z., J.W., and B.Z. performed molecular and cell experiments; W.Z. and X.T. performed bioinformatics analysis; Q.W., S.L., and M.L. analyzed data; Q.W. and S.L. wrote the manuscript. H.W., Y.Y., W.Z., and X.J. verified the data. All authors have read and agreed to the published version of the manuscript.

## Ethics Approval

The study protocol conforms to the ethics guidelines of the Declaration of Helsinki. All protocols involving human sample collection and use were approved by the Ethics Committee of Union Hospital, Tongji Medical College, Huazhong University of Science and Technology (Approved No. 2024‐0640‐01).

All animal experiences were approved by the Animal Ethic committee of Renmin Hospital of Wuhan University (Approved No. WDRM‐20240601A).

## Consent to Participate

Informed consent was obtained from patients and/or their authorized agent before obtaining clinical samples.

## Supporting information



Supporting Information

Supporting Information

## Data Availability

The data that support the findings of this study are available from the corresponding author upon reasonable request.
