# Supplementary material for: TRIM56 Aggravates Cerebral Ischemia‐Reperfusion Injury via Inhibiting KLF4‐Activated Ferroptosis Signaling
Source: Adv Sci (Weinh). 2025 Nov 10;13(8):e09906. doi: 10.1002/advs.202509906 (PMC12884795; doi:10.1002/advs.202509906)
Supplement: Supplementary file 1 — Supporting Information [file ADVS-13-e09906-s002.pdf]

Supplementary Figure S1 Construction of TRIM56-KO mice.

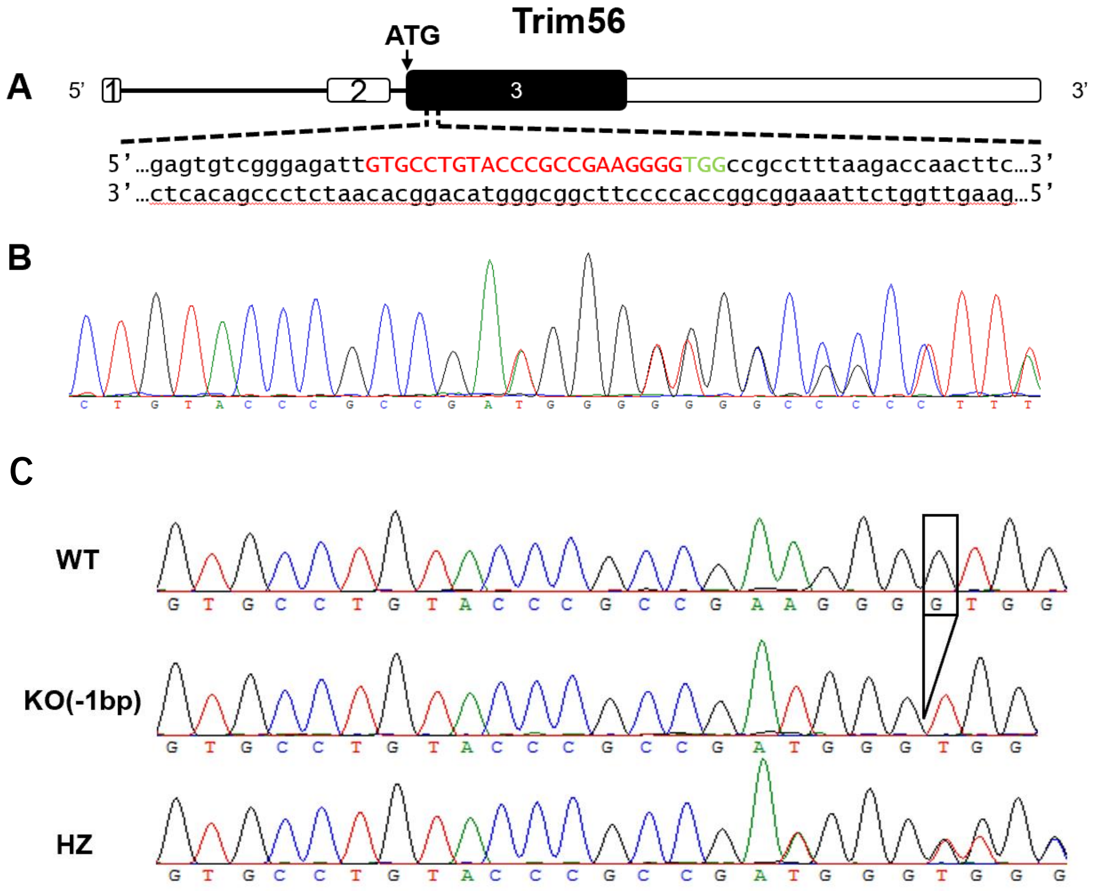

(A) Schematic of the C57/6J mouse *Trim56* gene. The translation start site (ATG) is located in E3, and the single-guide RNA (sgRNA) targeted site is highlighted in red. (B) Representative results from the DNA sequencing of founders. The sequencing chromatogram of heterozygous mutants revealed an indel that resulted in double peak traces. (C) DNA sequence chromatograms of *Trim56*-forward and *Trim56*-reverse PCR products illustrating the 1 bp deletion in the homozygous (*Trim56*$^{-/-}$) sample. Notes: WT: wild type; KO: knockout; HZ: heterozygous.

Supplementary Figure S2 The results of ferroptosis-related protein screening.

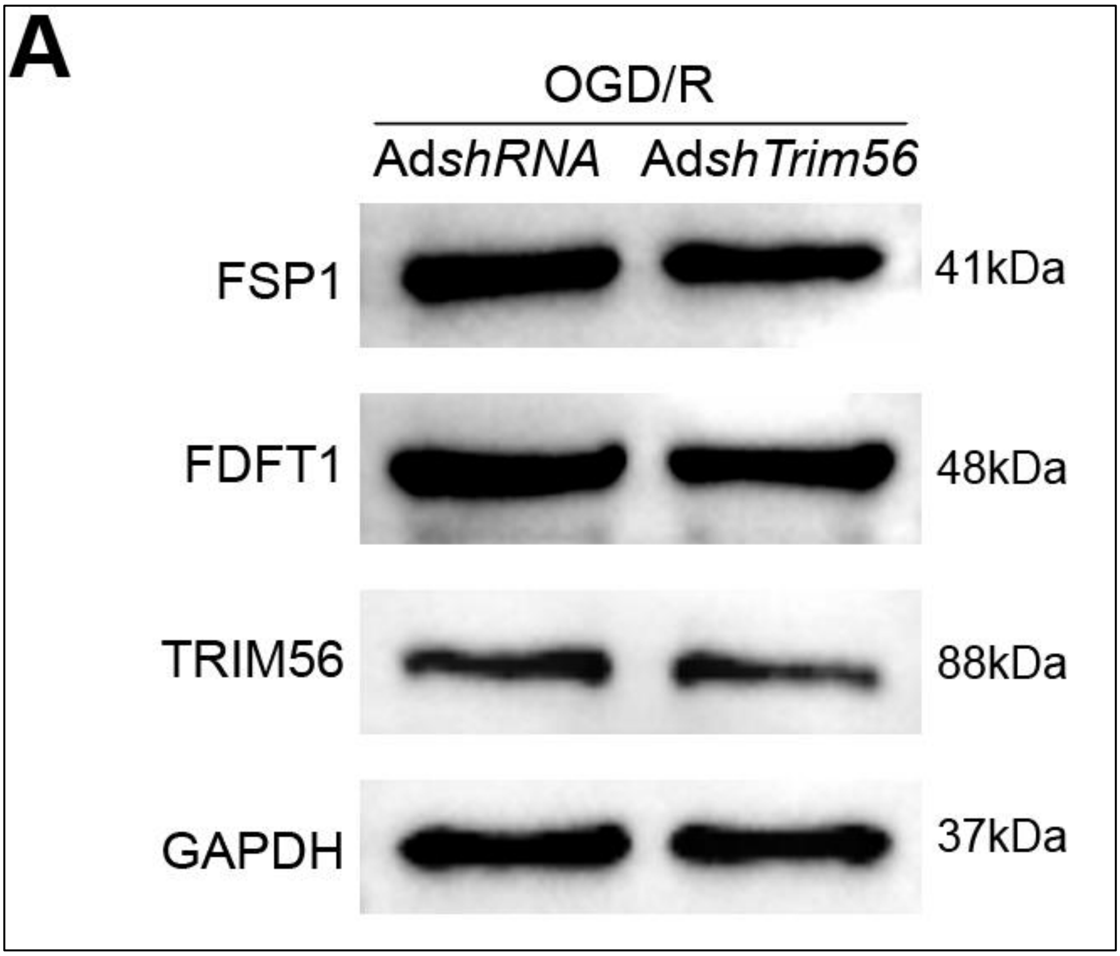

(A) Representative images of western blot of FSP1 and FDFT1 in primary neurons after OGD/R stimulation.

Supplementary Table S1 The clinical characteristics of human samples.

Variable	All cases	Control group	IS group	*p* value
No. of cases	9	3	6	N.A.
Mean age (SD)	58.89±4.56	59.67±4.04	58.50±5.20	0.963
No. of Male	5	2	3	0.684
NIHSS score	N.A.	N.A.	28.33±4.13	N.A.
WBC counts	11.58±3.05	8.85±3.42	12.94±1.88	0.048
NEUT (%)	84.11±11.79	72.17±13.85	90.08±4.17	0.017
Duration of				
Ischemia (hours)	N.A.	N.A.	5.50±1.87	N.A.
Reperfusion (days)	N.A.	N.A.	4.16±1.72	N.A.

Notes: IS: ischemic stroke; N.A.: not available; NIHSS: National Institute of Health stroke scale; WBC: white blood cell; NEUT: neutrophil percentage.

Supplementary Table S2 The primer sequences were used in qRT-PCR.

Gene names		Sequences (5'-3')
TRIM56 (Human)	Forward	CAGCTCTGGCTAGTTCTCACA
	Reverse	CCTTGGGCCACTTGCTCTT
GAPDH (Human)	Forward	CATCACCATCTTCCAGGAGCGAGA
	Reverse	TGCAGGAGGCATTGCTGATGATCT
Trim56 (Mouse)	Forward	CTCCCCAACTCTGCTGGAAG
	Reverse	TGACCACCGATGTCCAGTTG
Tnf-α (Mouse)	Forward	TTCTATGGCCCAGACCCTCA
	Reverse	AAGGTACAACCCATCGGCTG
Il-6 (Mouse)	Forward	TAGTCCTTCCTACCCCAATTTCC
	Reverse	TTGGTCCTTAGCCACTCCTTC
Il-1β (Mouse)	Forward	CCGTGGACCTTCCAGGATGA
	Reverse	GGGAACGTCACACACCAGCA
Ccl2 (Mouse)	Forward	TACAAGAGGATCACCAGCAGC
	Reverse	ACCTTAGGGCAGATGCAGTT
Cxcl2 (Mouse)	Forward	GCGCCCAGACAGAAGTCATA
	Reverse	CAGTTAGCCTTGCCTTTGTTCA
KLF4 (Mouse)	Forward	TACCCCTACACTGAGTCCCG
	Reverse	GAAAGGAGGGTAGTTGGGCC
GAPDH (Mouse)	Forward	ACTCCACTCACGGCAAATTC
	Reverse	TCTCCATGGTGGTGAAGACA
Trim56 (Rat)	Forward	TCCCGCTGGGAGAATCCAA
	Reverse	GAGACTTTGGAGGTCATACTGGA
Tnf-α (Rat)	Forward	ATGGGCTCCCTCTCATCAGT
	Reverse	GCTTGGTGGTTTGCTACGAC
IL-6 (Rat)	Forward	CCCAACTTCCAATGCTCTCCT
	Reverse	TAGCACACTAGGTTTGCCGA
IL-1β (Rat)	Forward	GACTTCACCATGGAACCCGT
	Reverse	CAGGGAGGGAAACACACGTT

Cxcl10 (Rat)	Forward	TGAAAGCGGTGAGCCAAAGA
	Reverse	CTAGCCGCACACTGGGTAAA
KLF4 (Rat)	Forward	GAAGGGAGAAGACACTGCGT
	Reverse	GCTCCACCACTTTCCAGGTC
GAPDH (Rat)	Forward	CAGTGCCAGCCTCGTCTCAT
	Reverse	AGGGGCATCCACAGTCTTC

Supplementary Table S3 The antibodies were used in western blot.

**Antibody**	**Catalogue number**	**Manufacturer**	**Source**	**Dilution**
Trim56	Ab154862	abcam	rabbit	1:1000
FSP1	A12128	ABclonal	rabbit	1:1000
FTFD1	A6229	ABclonal	rabbit	1:1000
4-HNE	A26085	ABclonal	rabbit	1:1000
IKKβ	A0714	ABclonal	rabbit	1:1000
p-IKKβ	2697	CST	rabbit	1:1000
IκBa	4814	CST	mouse	1:1000
p65	8242	CST	rabbit	1:1000
p-p65	3033	CST	rabbit	1:1000
Flag	M185-3L	MBL	mouse	1:1000
xCT (Slc7a11)	A2413	ABclonal	rabbit	1:1000
GPX4	A25009	ABclonal	rabbit	1:1000
TXNRD1	A4725	ABclonal	rabbit	1:1000
KLF4	A13673	ABclonal	rabbit	1:1000
HA	M132-3	MBL	mouse	1:1000
Myc	M192-3	MBL	mouse	1:1000
GAPDH	60004-1-lg	Proteintech	mouse	1:50000
