# Supplementary figures and images for: TRIM56 Aggravates Cerebral Ischemia‐Reperfusion Injury via Inhibiting KLF4‐Activated Ferroptosis Signaling
Source: Adv Sci (Weinh). 2025 Nov 10;13(8):e09906. doi: 10.1002/advs.202509906 (PMC12884795; doi:10.1002/advs.202509906)

1I

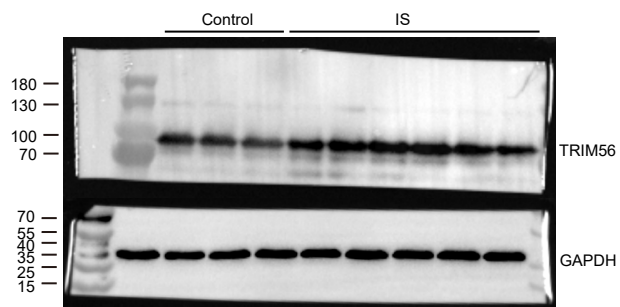

1Q

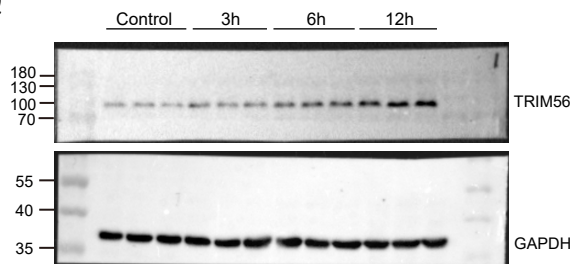

1L

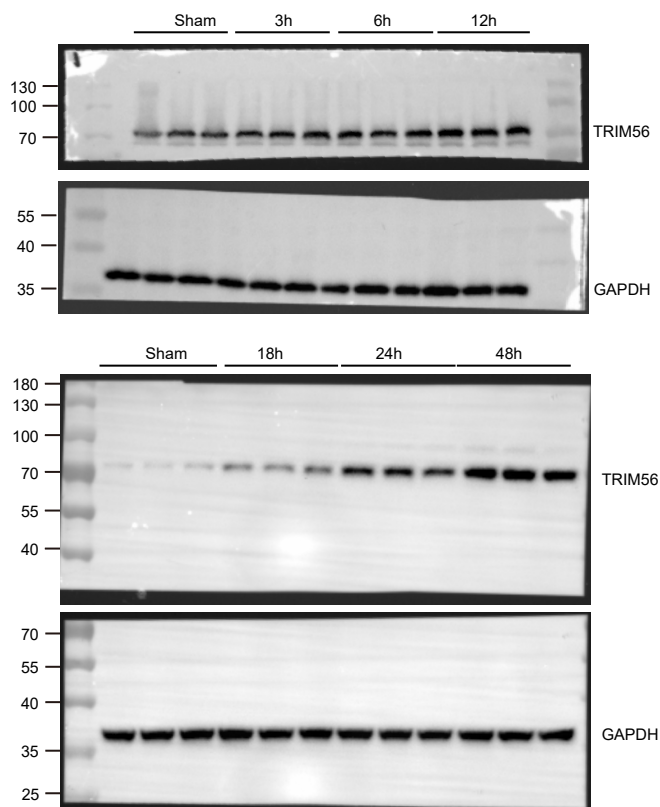

2A

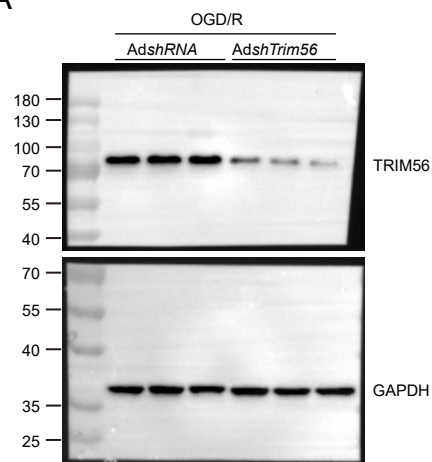

2D

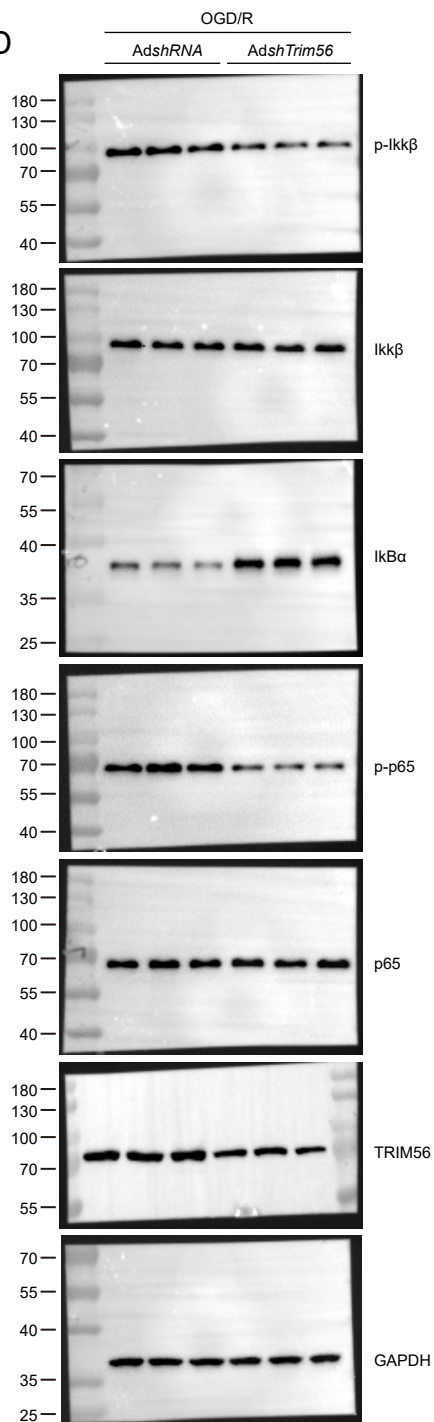

3A

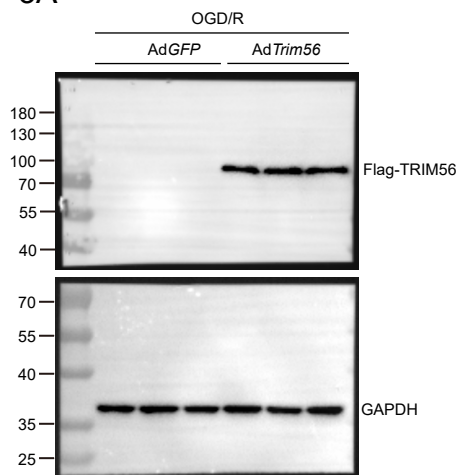

3D

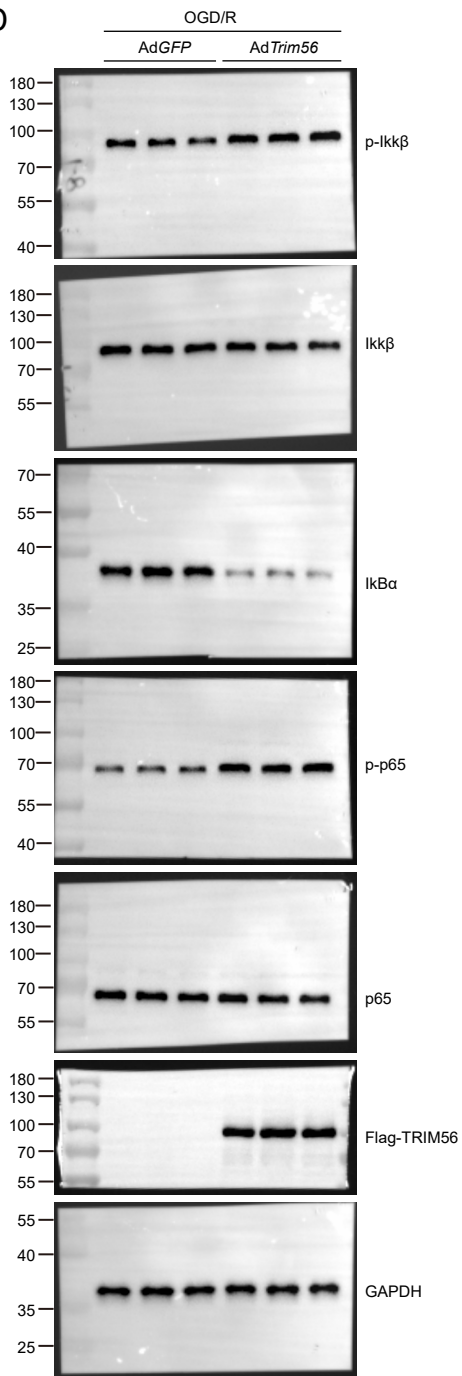

4A

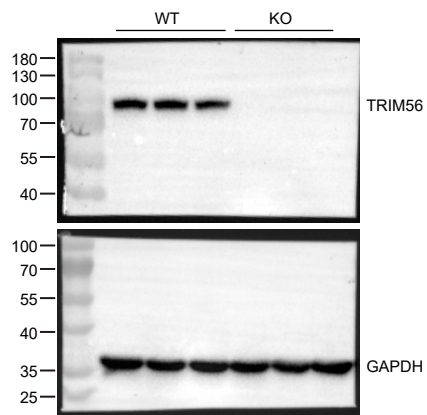

4G

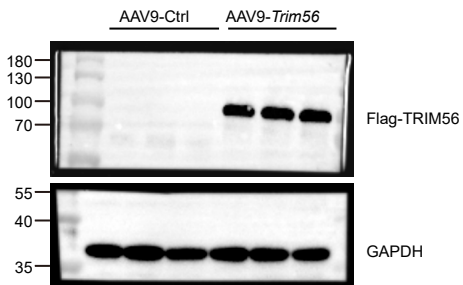

5E

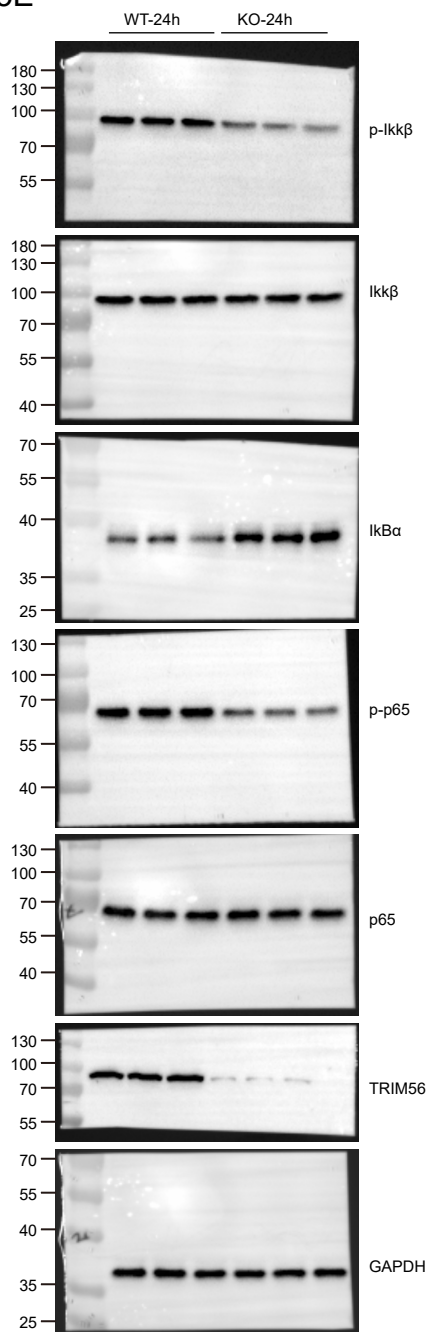

6J

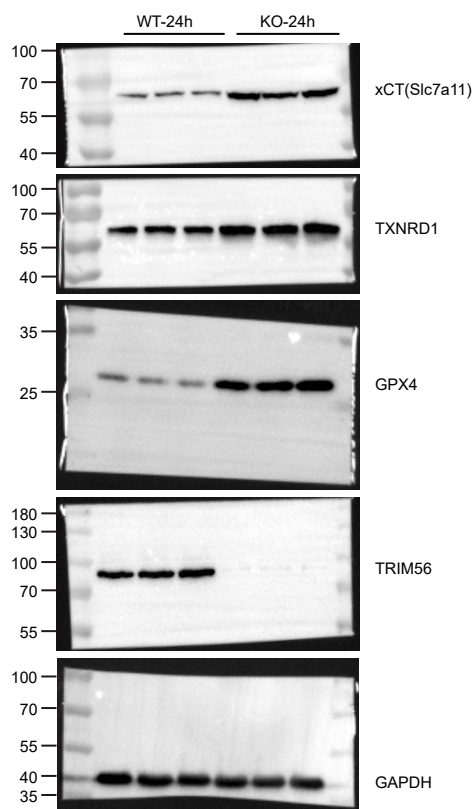

6M

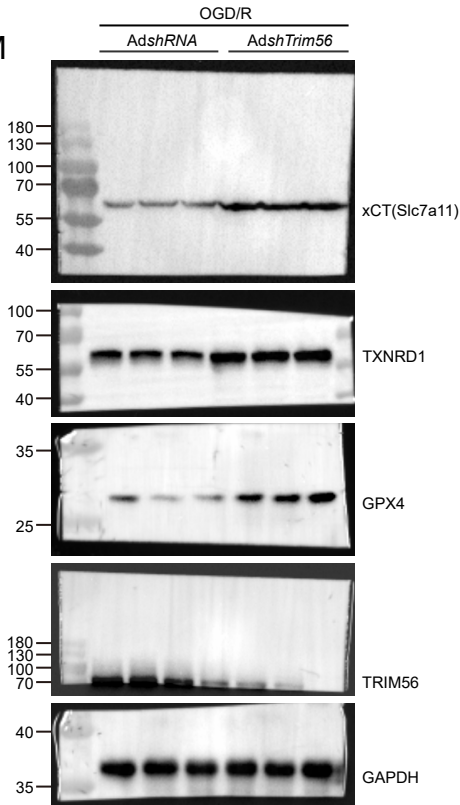

6P

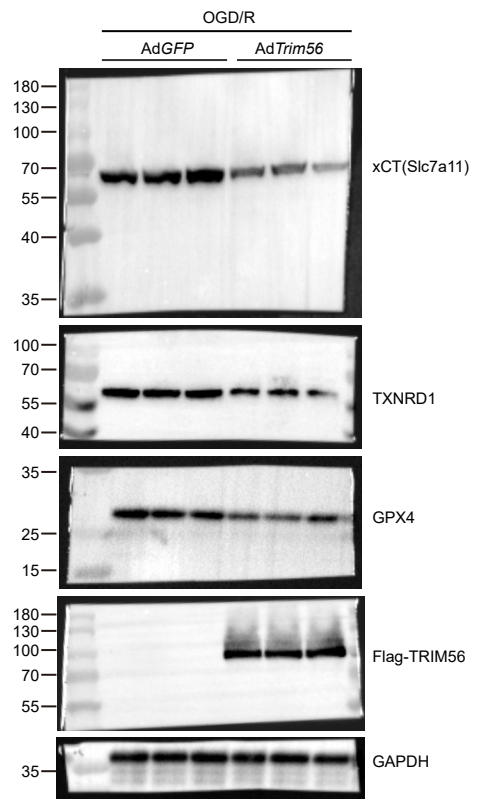

7D

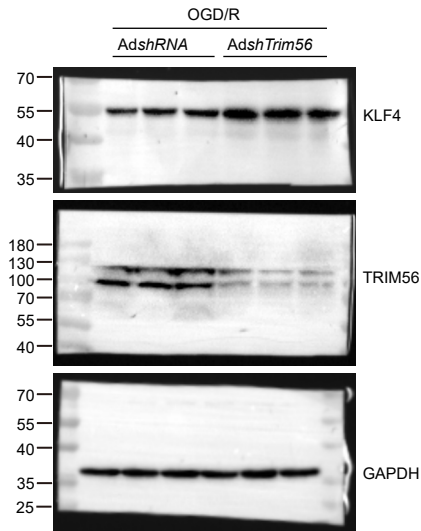

7F

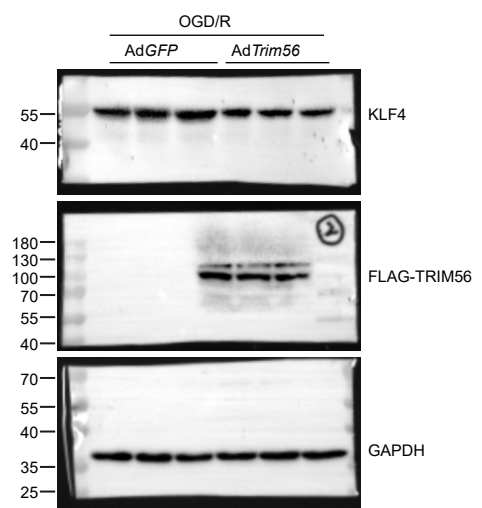

7G

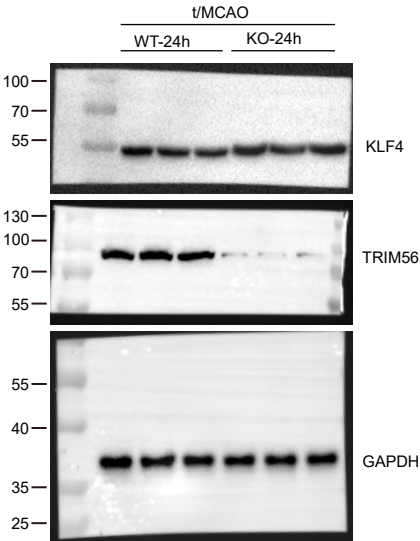

7H

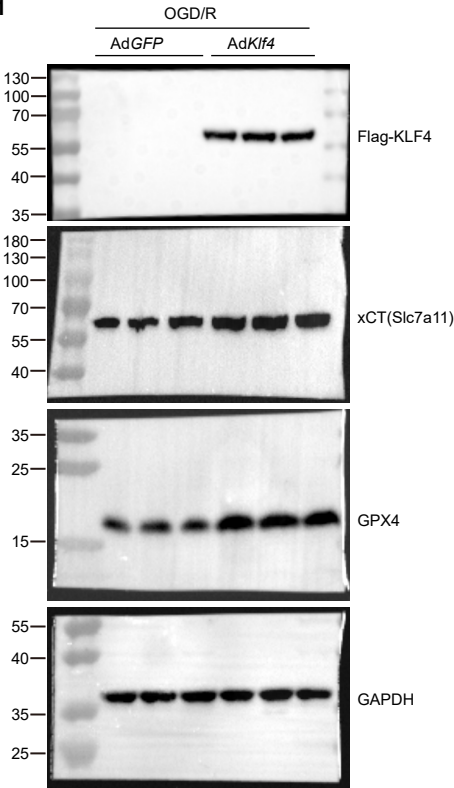

7I

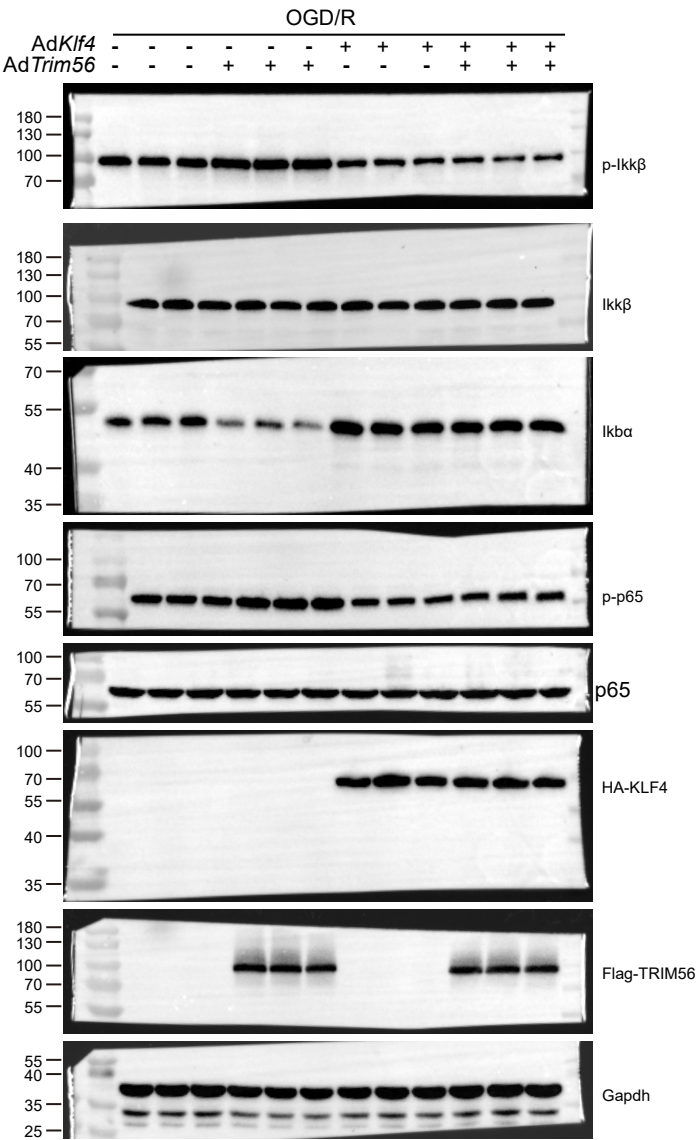

7J

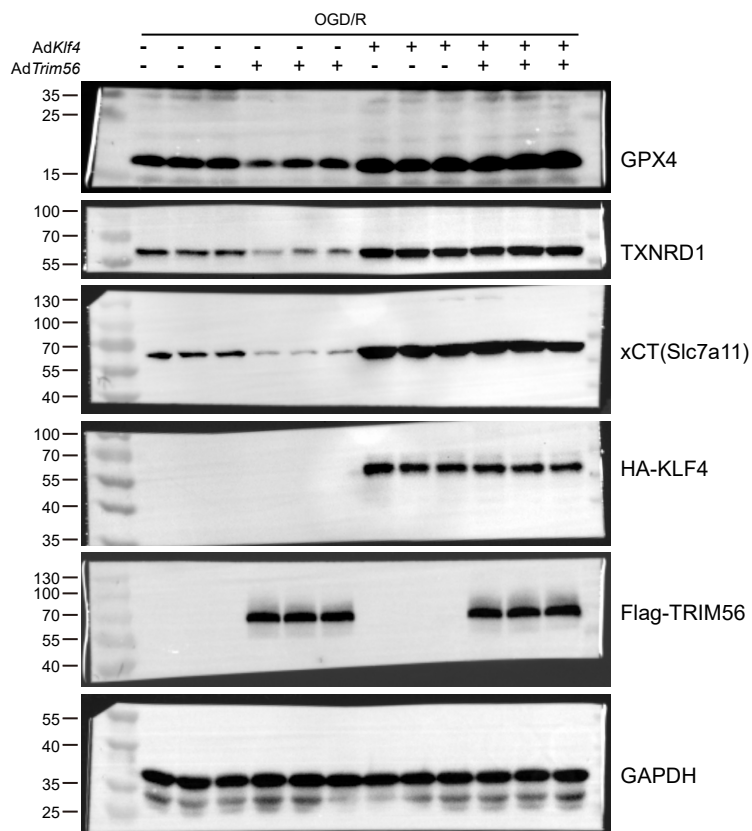

8C

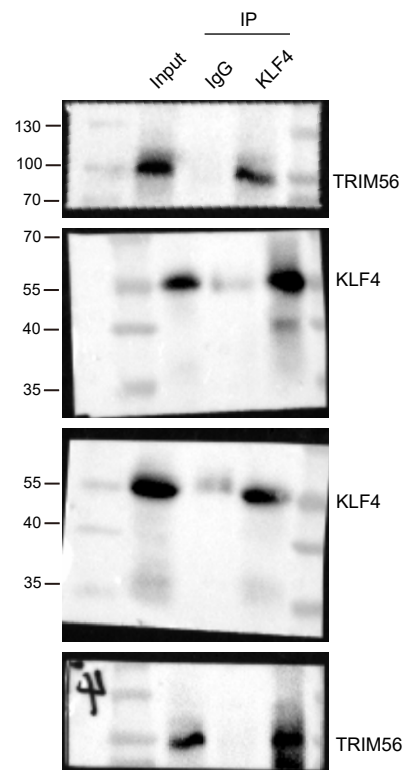

8D-1

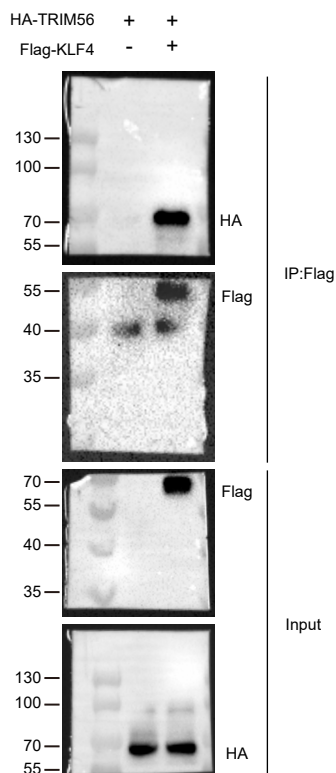

8D-2

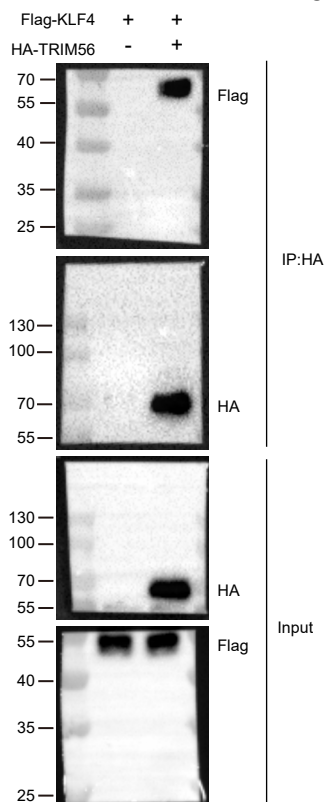

8E

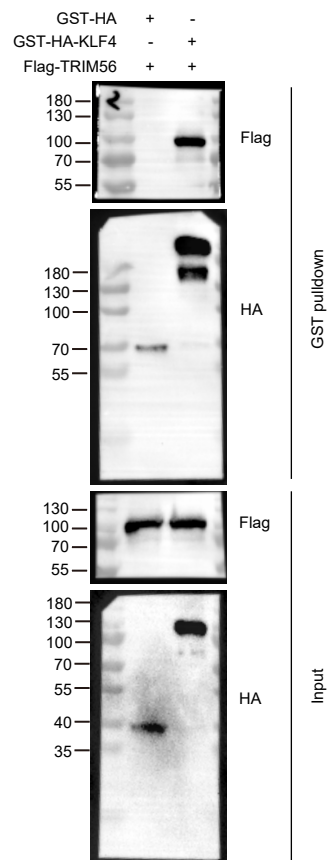

8F

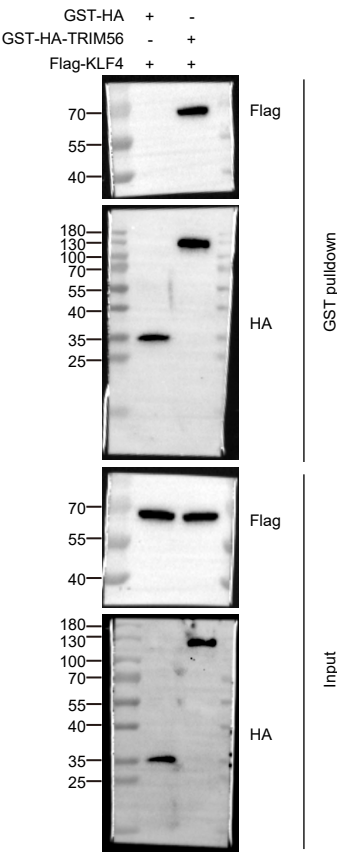

8G

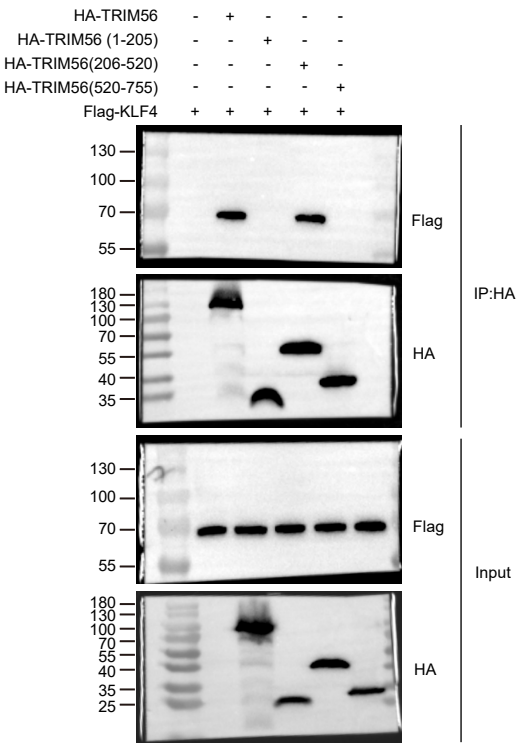

8H

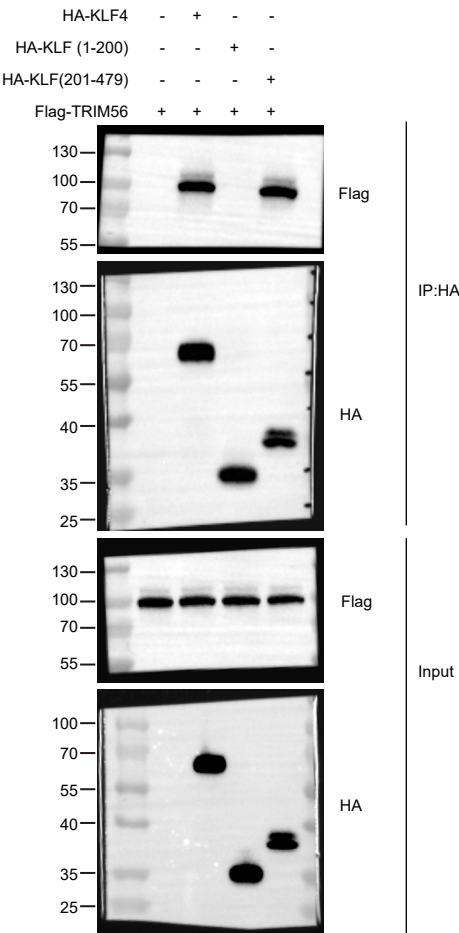

8I

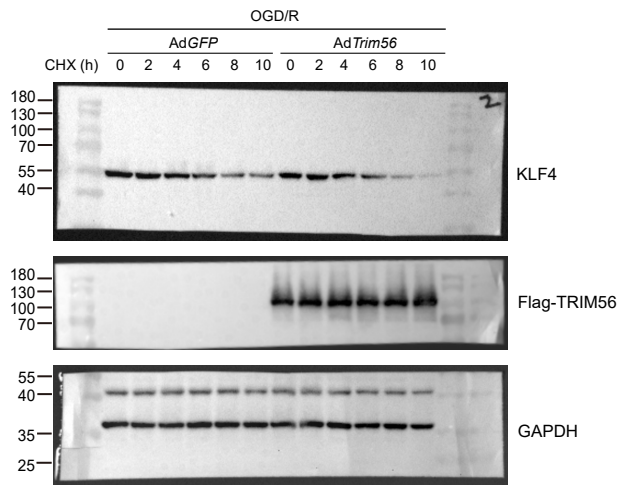

8J

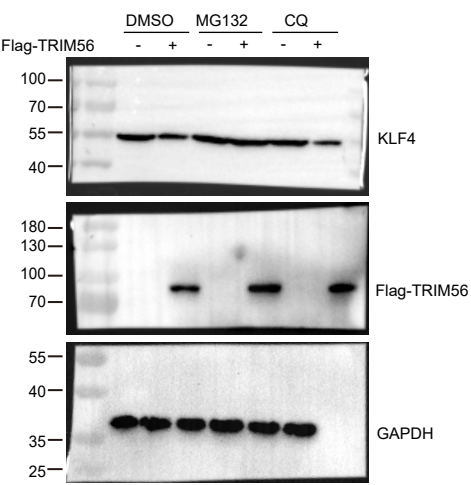

8K

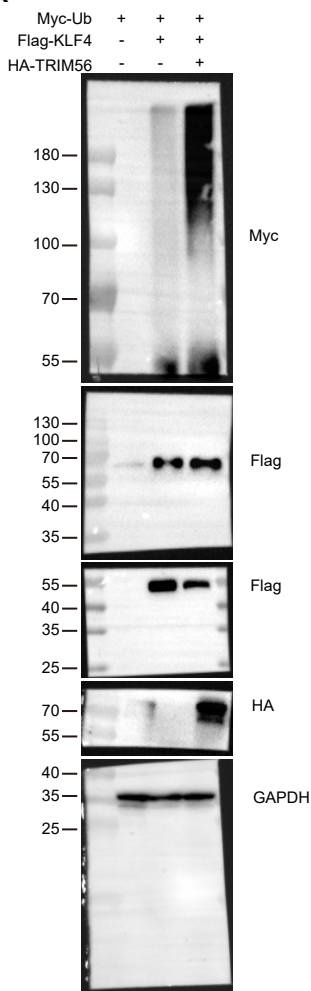

8L

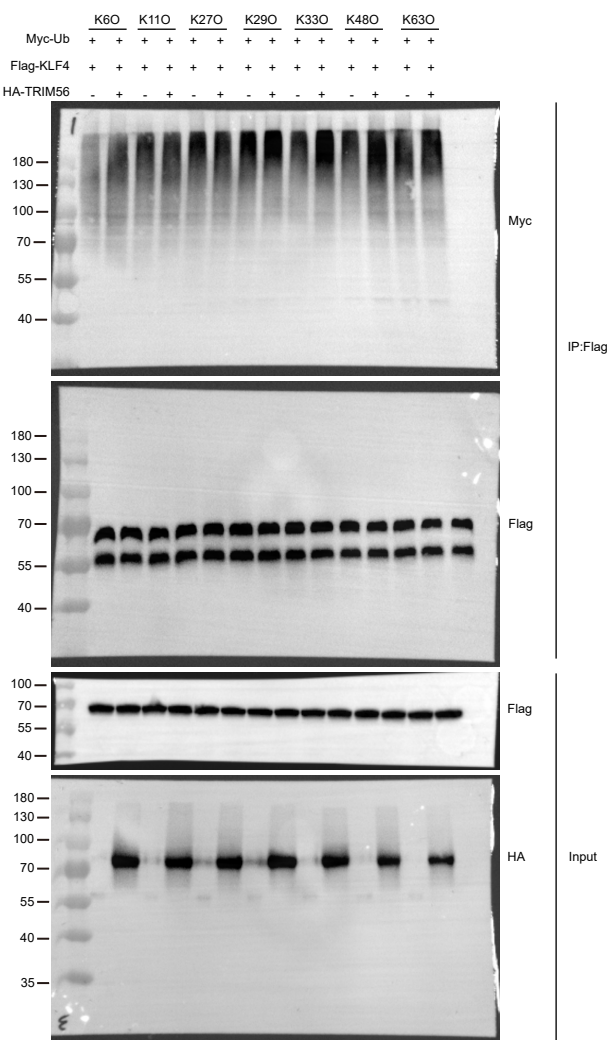

9F

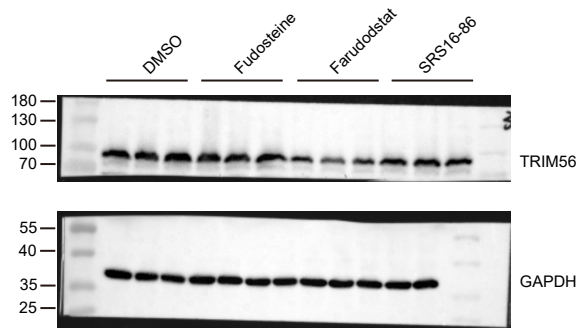

9K-2

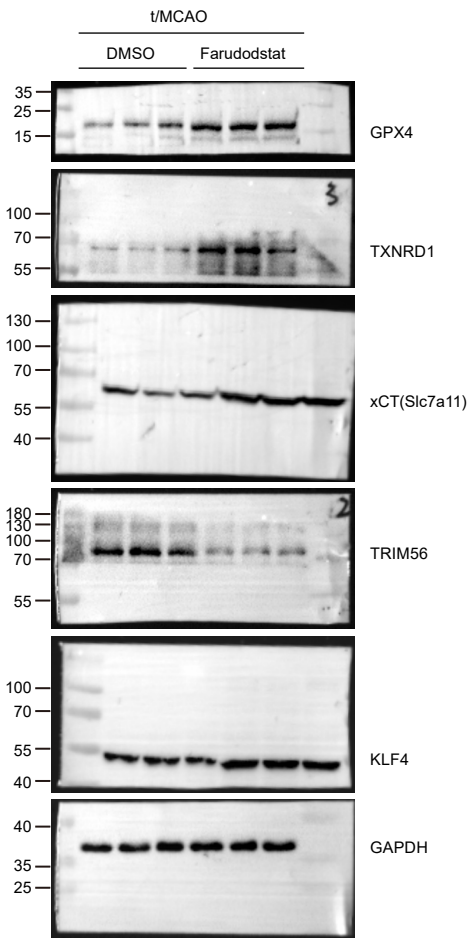

9K-1

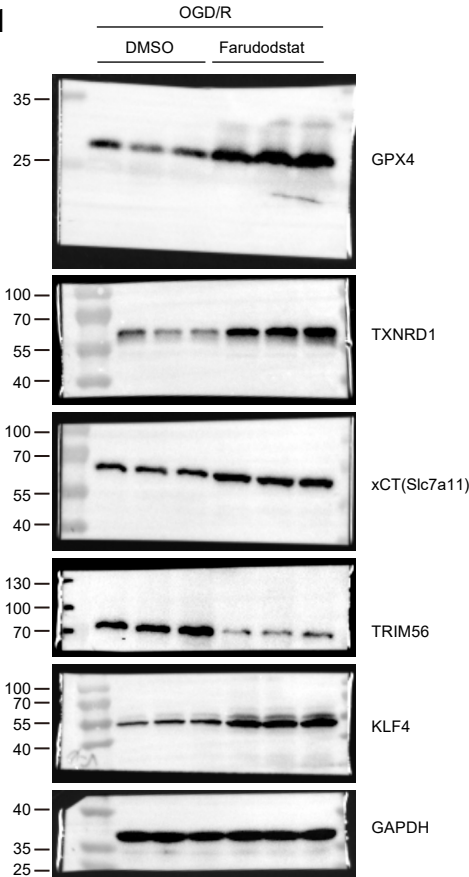

9M

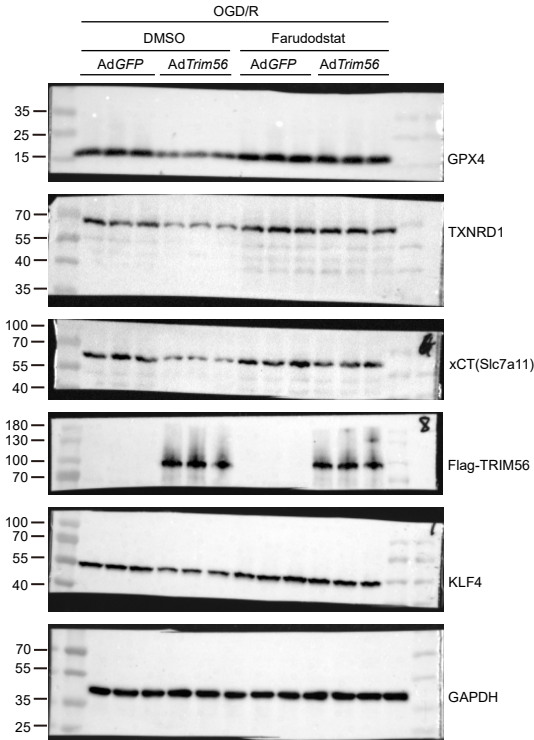

S2A

OGD/R  
*AdshRNA* *AdshTrim56*

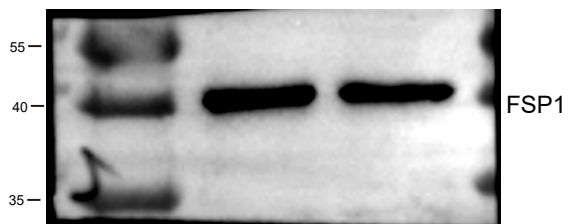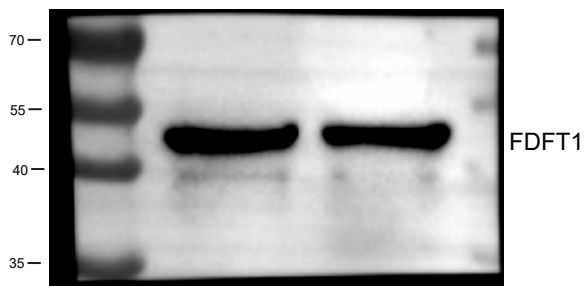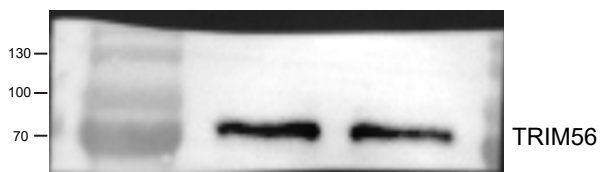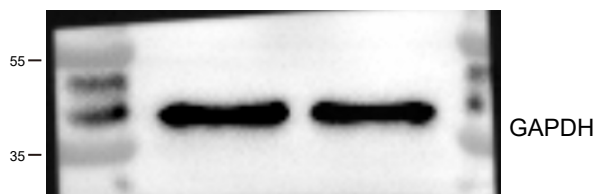

Supplement: Supplementary file 2 — Supporting Information [file ADVS-13-e09906-s001.pdf]
